# Materials and Fabrication of Photocatalytic Hollow Fibers and Hollow Fiber Membranes

**DOI:** 10.3390/ma19143024

**Published:** 2026-07-14

**Authors:** Chrysoula Athanasekou

**Affiliations:** Institute of Nanoscience and Nanotechnology, National Center for Scientific Research “Demokritos”, Agia Paraskevi, 15341 Athens, Greece; c.athanasekou@inn.demokritos.gr

**Keywords:** photocatalyst, hollow fibers (HFs), hollow fiber membranes (HFMs), mixed matrix, external coating, spinning process, phase inversion process

## Abstract

Photocatalysis is widely used for toxic air and water pollutant degradation, water slitting and H_2_ production, and the reduction of CO_2_ to useful hydrocarbons. Hollow fibers (HFs) have been widely used as photocatalyst immobilizers for advanced oxidation and reduction applications under batch conditions, with active semiconductors either applied on their surface or incorporated into their matrix. Photocatalytic hollow fiber membranes (HFMs), the porous version of the above-mentioned fibers, which exhibit dual functionality in the degradation and physical separation of contaminants, are currently applied under flow conditions for wastewater recycling and reuse. This work provides a concise overview of all the studies encountered in the literature on photocatalytic HFs and HFMs, categorizes them with respect to their materials and fabrication methods and aspires to serve as a guide for anyone wanting to prepare and use them in photocatalytic batch or flow reactors.

## 1. Introduction

In recent decades, the literature has been overflowing with scientific publications concerning photocatalysis. Photocatalysts are transition metal oxide semiconductors that can be activated when irradiated and accelerate chemical reactions. They possess the ability to absorb energy and create charge carriers within their own body that can travel to the surface and hit any molecules within reach. These, in turn, may be H_2_O molecules or organic or inorganic molecules in the aquatic or gas phase. This family of molecules consists, principally, of metal oxide powders, with TiO_2_ holding the chief position. The solid nature of photocatalysts categorizes photocatalysis as heterogeneous catalysis, with the final separation of the suspended catalyst powder from the aquatic media being its main problem.

**Figure 1:**
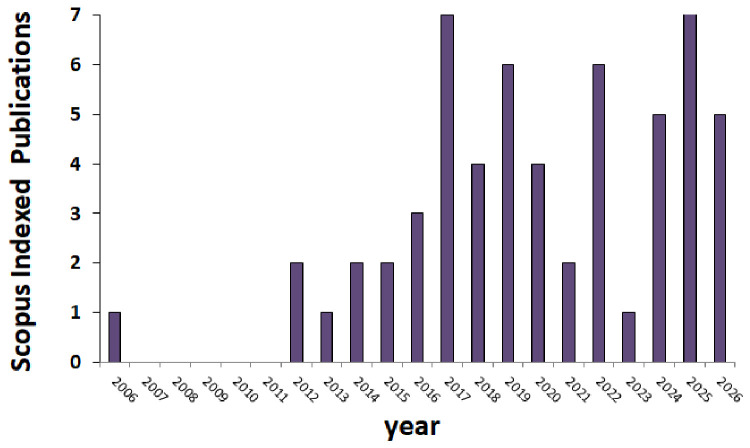
The graphical bibliographic visualization of the Scopus-indexed publications on photocatalytic HFs per year.

To overcome this problem, the immobilization of photocatalysts on easy-to-recycle elements has been widely studied, with hollow fibers (HFs) being an often-encountered representative. Their advantageous configuration, offering a high surface area with increased potential interface reaction sites, together with the benefits from light harvesting and reflecting hollowness, makes them excellent candidates for photocatalyst immobilization. The growth in research interest and publication trends in this field throughout the years until the first half of 2026 is depicted in Figure 1. The graphical bibliographic visualization includes only cases of HFs with immobilized catalysts, the photocatalytic application of which is clearly described within this paper.

The photocatalytic applications of HFs refer mainly, but not exclusively, to wastewater treatment, being tested against model pollutants, e.g., azo-dyes, pesticides, pharmaceuticals, or endocrine disruptive compounds. Specifically, methylene blue (MB) is the most studied dye [1,2,3,4,5,6,7,8,9,10,11], followed by methyl orange (MO) [12,13,14,15], Congo red (CR) [10] and Rhodamine B (Rhd B) [16]. Bisphenol A, a chemical compound used in the production of plastics, suggested to cause metabolic kinetic alterations and chromosomal and DNA damage, has also been tested [10,17,18,19,20,21,22,23,24,25], along with other endocrine disruptors, including nonyphenol (NP) [26],25] and phenol [10]; the pharmaceuticals diphenhydramine (DP) [27], gabapentin [25] and diclofenac [25]; caffeine [25] and pesticides [28,29,30]. COD was also tested [31].

A subset of photocatalytic HFs consists of photocatalytic hollow fiber membranes (HFMs). Although HFs may or may not be porous, depending on their fabrication method, HFMs always possess a structural porous network. Therefore, when they are applied under flow conditions, they can perform photocatalysis and filtration in one step, separating one or more of the reaction’s products from the reaction mixture. Concerning HFs and HFMs, confusion is encountered in the literature regarding the following: when HFs occur from a phase inversion process, they unavoidably possess pores, and, therefore, many authors identify their HFs as HFMs only because they are porous. This is misleading. HFs cannot be referred to, in general, as “membranes”, unless they are applied as filters (under flow conditions) [32]. If the application they are used in is a batch one, and they perform solely as photocatalyst immobilizers, they should be called HFs, although they may possess a porous network.

Among articles that actually employ HFs not only in batch reactors but in actual flow reactors as HFMs, the degradation of MB [33,34], acid orange 7 (AO7) [35,36] and NP [37] is encountered, along with plenty of antifouling/membrane self-cleaning applications [35,36,37,38,39,40,41,42,43]. Apart from wastewater treatment, HFs and HFMs have also been utilized in gas-phase photocatalytic applications, namely, the degradation of ammonia [44], acetaldehyde [45] and formaldehyde [1]; the reduction of NO [46,47]; and the production of H_2_ [16,48].

TiO_2_ is the most widely used photocatalyst, encountered commercially in mixtures of anatase (70–80%) and rutile (30–20%); it possesses photocatalytically active and thermodynamically stable tetragonal crystalline forms, respectively, with its exceptional activeness being attributed to a synergic effect between them. As expected, the majority of the studies mentioned have used pure TiO_2_ or TiO_2_-based heterostructures [1,2,3,4,6,7,8,9,10,11,12,13,14,15,16,17,18,19,22,23,26,27,28,29,30,31,32,33,34,35,36,37,39,40,41,42]. The only exceptions are those of Nb_2_O_5_ [5], Cu_2_O [20,24], WO_3_ [34,38], CuO–VS_4_ [21], MoO_3_/ZnO/GO [25], CuO/CeO_2_ [38], Zn_2_SnO_4_ [10] and g-C_3_N_4_ [34,39]. The wide band gap (3.2 eV) of TiO_2_ requires UV light activation, which represents only 4% of solar light [49], with a lot of studies, therefore, trying to transfer its required activation energy to the visible light area [50,51]. Among photocatalytic HFs, studies using visible light are limited to N-doped [19,41,42,44], Fe-doped [3,45], Au-doped [4], S-doped [43] and Ag-doped [22,23] TiO_2_; TiO_2_-WO_3_@GO photocatalysts [40]; and TiO_2_/carbon nanocomposites (GO, C_60_, and CNTs) [27,48]. Other visible light photocatalysts used are CuO–VS_4_ [21], Zn_2_SnO_4_ [10], In_2_O_3_/ZnIn_2_S_4_ [48] and Cu_2_O [20,24], with attempts close to visible light being those using xenon lamps for simulated solar light [2,9,16,22,23,34,43,46,48].

The present study summarizes all the materials used and the fabrication routes followed to produce the photocatalytic HFs and HFMs encountered in the literature. It specifies the relation between the fabrication method and the potential to distinguish between HFs and HFMs, and clarifies the misunderstanding encountered in the literature around this topic. As photocatalysis is currently a trend in toxic compound degradation and wastewater treatment, this review aspires to serve as a guide in the field.

## 2. Mixed Matrix Photocatalytic HFs and HFMs

### 2.1. Conventional Spinning–Extrusion–Sintering Fabrication

**Figure 2:**
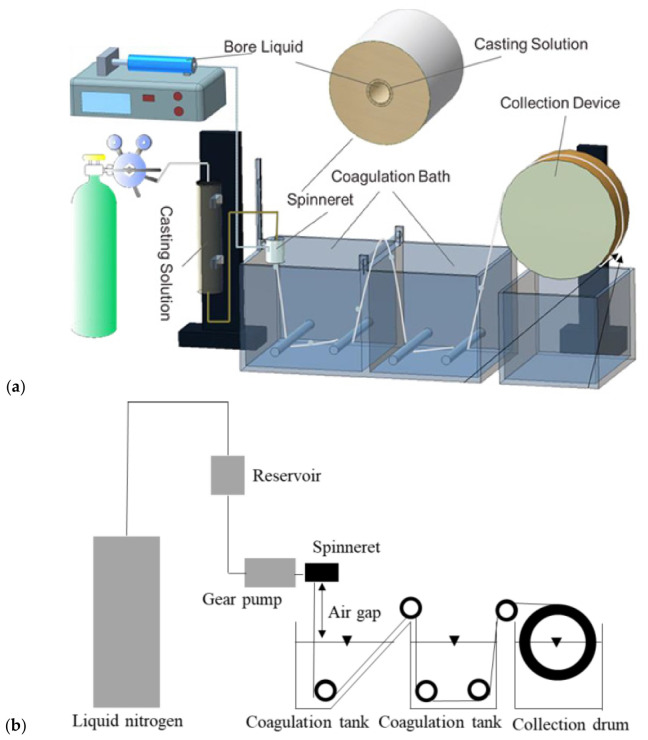
(**a**) Illustration of the wet (wet/wet) spinning setup (reprinted with permission from Ref. [41]. Copyright year 2006, American Chemical Society). (**b**) Illustration of the dry/wet spinning setup (reprinted from open access reference [52]; no permission needed).

The most common way to produce photocatalytic HFs is through a spinning process with the photocatalyst immersed in a polymer blend and extruded through a spinneret orifice. The technique is categorized as dry, wet (often encountered as wet/wet), or dry/wet, depending on how the polymer precipitates to form the fiber. In dry spinning, solvent evaporates with hot air; in wet spinning, the polymer precipitates in a liquid bath (Figure 2a) and the hybrid dry–wet extrudes polymer into a brief air gap (Figure 2b) before entering the coagulation bath (nonsolvent) for precipitation. The hollow structure of the fibers is attributed to the coextrusion of the polymer solution simultaneously with a bore fluid, via special concentric orifice spinnerets. The bore fluid (Figure 2a) consists of a polymer’s bad solvent (usually water). The process is primarily a dry–wet one, which involves extruding firstly into air (Figure 2b) and then into a coagulation bath, allowing for better control of the fiber properties and faster speeds. As mentioned above, the identifying element that distinguishes an HF from an HFM is the porous structure, enabling it with the separating/filtrating potential. This special structural characteristic is being given to the newly produced hollow fibers through phase inversion in the nonsolvent (for the polymer) bath, creating membranes with controlled pores for filtration/separation uses. Mixed matrix photocatalytic HFs and HFMs have been synthesized using the following polymers.

#### 2.1.1. Alginate Biopolymer

The very first polymer reported to have been employed for photocatalytic HF fabrication back in 2012 [12] was the alginate biopolymer, a water-soluble polysaccharide derived from brown algae. This linear block copolymer can form 3D networks in the presence of divalent cations, being able to stabilize, therefore, any photocatalyst. The biopolymer/Degussa P25 suspension was extruded, simultaneously with the bore liquid, directly into the 10% CaCl_2_ coagulation bath. Through this wet/wet spinning process, TiO_2_ was dispersed and stabilized into a hydrogel Ca-alginate HF matrix (200 mm ID, 500 mm OD) at a concentration of 3.6 g/L. Further treatment of this nonporous hydrogel fiber under supercritical CO_2_ conditions (100 bars, 45 °C), led to its highly porous aerogel form. The drying procedure consisted of exchanging the water contained in the fibers with ethanol in ethanol–water baths of increasing alcohol concentration (10–100%) for 30 min each and then removing ethanol with sc-CO_2_. The 31 cm^2^ HF surface of the porous Ca-alginate-TiO_2_ HFs achieved a 90% photocatalytic degradation of a 20 mM MO solution within only 220 min, while their nonporous analogs within 325 min.

Alginate biopolymer was also used as an immobilizer of nanocarbon–TiO_2_ composite photocatalysts. Carbon nanotubes (CNTs), fullerenes (C_60_), and graphene oxide (GO) were used as additives of Degussa P25 in percentages between 4 and 12% into HFs [27], targeting beneficial electronic properties against DP and MO in batch conditions. In both cases, 4 wt.% GO/TiO_2_ proved best, with reaction rates k = 7.7 × 10^−3^ min^−1^ (MO) and k = 3.4 × 10^−3^ min^−1^ (DP).

Years later [13,14] the same alginate/TiO_2_ HFs were decorated with copper nanoparticles in the state of metallic and CuO, by meeting copper nitrate (1 g Cu(NO_3_)_2_·3H_2_O in 100 mL of EtOH) for 24 h. This incorporation of either carbon nanocomposites or copper nanoparticles proved catalytical for the transition of the photocatalyst activation from the UV to the visible light area, enabling it with a more sustainable effectiveness. Therefore, they resulted in a 65.3% decolorization of an O_2_ saturated 12 mg/L MO solution within 3 h and 73.5% of a 6.3 mg/L MO solution within 3 h, with the photocatalyst maintaining 92% of its initial performance after 5 five successive cycles, at concentrations up to 15 mg/L.

Although not using a typical spinning device, Zhang et al. [46] prepared mixed matrix alginate HFs through a microfluidic device. The CNT/TiO_2_ in alginate/PVP solution (middle phase) and the calcium chloride solution (inner phase) were driven by a pump, with rates varying from 100 mL/h to 240 mL/h, through a coaxial needle directly into the outer phase solution. The wall thickness of the HFs was 60–150 μm, and their diameter was around 1000 μm. Here, 30 mg of the 10% CNT/TiO_2_ catalyst under a 350 W xenon lamp reduced 44.3% of a 200 ppm NO.

#### 2.1.2. Polyether Imide (PEI)

PEI has been the carbon source for the preparation of photocatalytic carbon/TiO_2_ HFs. The spinning dope was created by mixing PEI/P25 TiO_2_ nanoparticles (Evonik) and NMP solvent (1-methyl-2-pyrrolidinone) in the ratio of 18:25:75 (*w*/*w*) [35]. Phase inversion was induced from the inner side of the HF by introducing DI water at the rate of 40 mL/min and was completed by leaving the HF immersed in DI water for 24 h. The spinneret’s diameters were OD = 2.5 mm and ID = 0.8 mm, the pressure in the spinning dope was 4 bars, and the air gap between the spinneret and the coagulation bath was 50 mm. The extruded TiO_2_/PEI HF was calcined at a temperature in the range of 700 to 1400 °C for 8 h with a heating/cooling rate of 5 °C/min, and pure TiO_2_ HFMs were obtained as PEI was removed due to its pyrolysis during calcination. The same process was reported [36] with the pyrolysis taking place at 550 °C for 3–12 h in a muffle furnace without any special gas, and PEI gradually pyrolysed into char to the final composite char/TiO_2_. The as-prepared HFMs, although being membranes, were photocatalytically evaluated under batch conditions against AO7 as a model pollutant, the degradation of which reached the value of 66.7% in the presence of TiO_2_ calcinated at 700 °C and decreased to 41.5%, 25.1%, and 2.5%, with the increment of the calcination temperatures to 800, 900, and 1400 °C, respectively.

#### 2.1.3. Poly(m-phenylene isophthalamide) (PMIA)

A recent work [33] developed poly(m-phenylene isophthalamide) (PMIA) HFMs incorporating 0.00–6.25 wt.% TiO_2_ nanoparticles with 25 nm average particle size, by dispersing them uniformly in DMAc (ultrasonication 30 min) and adding them to a 17.5 wt.% PMIA solution. A 2 h stirring ensured homogeneous doping solution, which was then transferred to a spinning reservoir and degassed overnight to eliminate entrapped air. The spinneret’s OD/ID was 1.2/0.3 mm, with the flow rates of dope and bore fluid (DI water) to be 8 and 5 mL/min, respectively. The air gap distance was 10 cm. The extruded fibers upon contact with a nonsolvent coagulation bath underwent phase separation, leading to solidification and the formation of porous structures. The following 24 h soaking in a 30 wt.% glycerol solution was a pore-preserving treatment to prevent them from collapse. Concerning their application, the PMIA HFMs without TiO_2_ exhibited an initial water flux permeance of 207.5 L/m^2^h, which decreased when nanoparticles of TiO_2_ were incorporated and reduced their pore size. Under UV irradiation, the PMIA/TiO_2_ HFM exhibited improved pure water permeate flux, with a maximum flux of 132.6 L/m^2^h at 4.76 wt% TiO_2_, a behavior attributed to the photoinduced hydrophilicity of TiO_2_.

#### 2.1.4. Polyethersulfone (PES)

**Figure 3:**
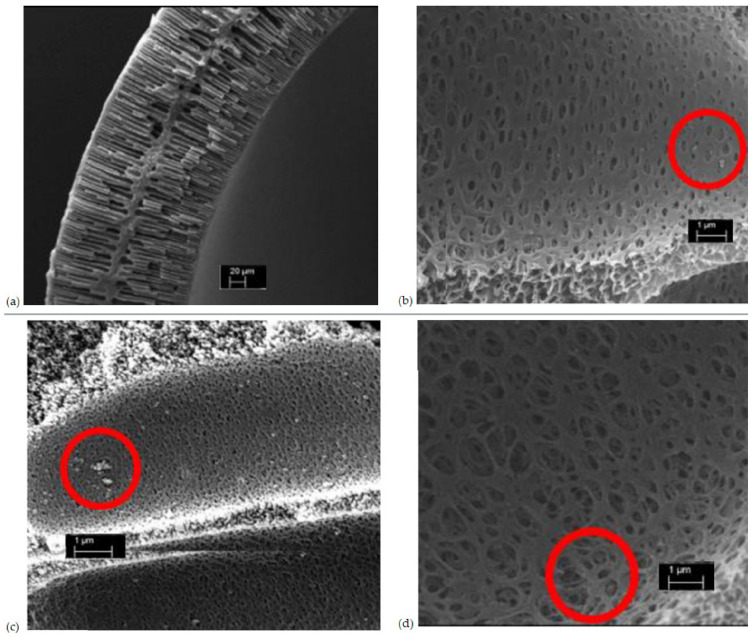
SEM pictures of the (**a**) PES HFs with optimized dope composition; cross-section 500×. The scale bar is 20 μm. (**b**) PES-TiO_2_ HFs. In the red circles, the 0.3% TiO_2_ NPS embedded in the matrix are highlighted. The scale bar is 1 μm. (**c**) PES-TiO_2_ HFs. In the red circles, the 0.5% TiO_2_ NPS embedded in the matrix are highlighted. The scale bar is 1 μm. (**d**) PES-TiO_2_ HFs. In the red circles, the 1% TiO_2_ NPS embedded in the matrix are highlighted. The scale bar is 1 μm (reproduced from open access Ref. [7]).

Another reported polymeric choice for photocatalytic HFs and HFMs is polyethersulfone (PES). PES exhibits high intrinsic hydrophilicity, due to its elevated percentage of sulfone groups, along with properties like processing ease, resistance, stability and compatibility with several additives such as photocatalysts. PES/TiO_2_ nanocomposite mixed matrix HFMs were prepared [7] with a comprehensive investigation of the role of additives in the polymer blend’s viscosity. Poly(ethylene glycol) (PEG) 400, Poly(vinyl pyrrolidone) (PVP), Pluronic^®^ F-127 (PEO–PPO–PEO), and Poly(ethylene glycol)-block-poly(propylene glycol)-block-poly(ethylene glycol) were employed as additives in various concentrations, with Pluronic being finally identified as the additive suitable for NMP solvent. The final dope was of TiO_2_ nanoparticles (NP) 0.3–1 wt%, with the photocatalyst source being a commercial suspension of TiO_2_-NPs in NMP with sizes between 60 and 100 nm. The newly produced HFMs were kept for 24 h in a frequently refreshed water bath for complete coagulation and residual solvent removal and soaked in 30% *w*/*w* glycerol to avoid porous structure collapse. Their cross-section and porous network are shown in the SEM pictures of Figure 3. Their photocatalytic activeness was tested only qualitatively (decolorization) under batch conditions employing 3 of the PES/TiO_2_ HFMs (20 cm each, total surface area 0.0036 m^2^) against MB (10 mg/L) under UV-A light (18 W, λmax at 365 nm) for 2 h at 0.6 mW/cm^2^.

**Figure 4:**
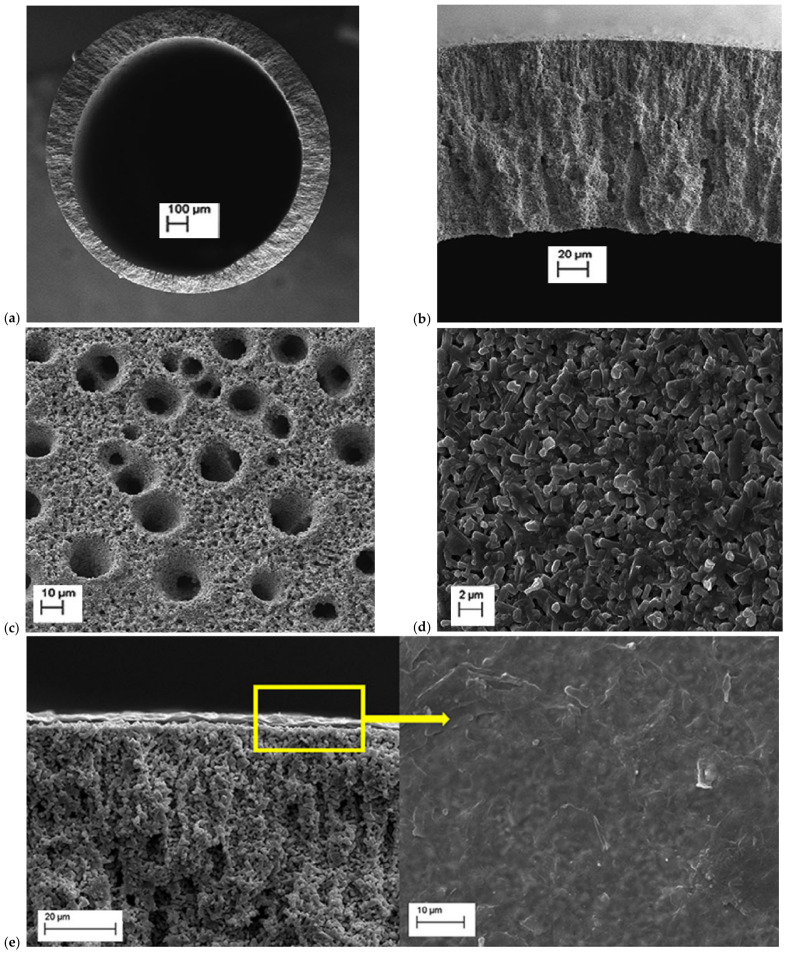
SEM images of the pristine Nb_2_O_5_ hollow fiber sintered at 1200 °C: (**a**) whole cross-section, (**b**) cross-section, (**c**) inner, and (**d**) outer surface views. (**e**) Cross-section and outer surface SEM images of the GO-coated Nb_2_O_5_ hollow fiber (reprinted with permission from Ref. [5], 2017 American Chemical Society).

PES was recently used for the fabrication of niobium pentoxide (Nb_2_O_5_) photocatalytic HFMs [5]. The primer suspension was prepared by dissolving 6 wt.% PES, 0.4 wt.% commercial additive polyethylene glycol 30-dipolyhydroxystearate (Arlacel P135, Croda, Snaith, UK), and 60 wt.% Nb_2_O_5_ (98.5%) powder in 33.6 wt.% dimethyl sulfoxide (DMSO) under stirring. After degassing, the ceramic suspension was extruded through an orifice spinneret (OD 3.0 mm, ID 1.2 mm (Figure 4a–d)) with dope and bore liquid (pure solvent DMSO) flow rates 10 and 15 mL/min, respectively, directly into a tap water bath and left to phase inversion process completion. Sintering took place at 1200 °C with a ramp of 2 °C/min (from room to 300 °C), held for 5 h, then with 1 °C/min (from 300 to 600 °C) and held for 1 h for polymer removal. The final temperature ramp was at the rate of 5 °C/min (from 600 to 1200 °C) with a dwelling time of 5 h. GO was deposited on the outer surface of the ceramic Nb_2_O_5_ HFs through vacuum-assisted (50 mmHg) dip coating in a GO 0.01 g/L aqueous suspension (Figure 4e). Although being HFMs, they were tested photocatalytically in batch experiments (as HFs) against 500 mL of 10 mg/L MB and pH = 11. HFs of 6 cm, containing 28.7 mg Nb_2_O_5_ each (catalyst of 57.4 mg/L), were irradiated for 210 min and achieved a 66.4% photo degradation efficiency, while the GO-coated Nb_2_O_5_ HFs had an efficiency of 100% and a successful reuse for 4 reaction cycles (240 min), showing long-term stability.

#### 2.1.5. Polyvinylidene Fluoride (PVDF)

Concerning the preparation of photocatalytic HFs and HFMs, polyvinylidene fluoride (PVDF) is among the leading choices, due to its elevated chemical resistance and oxidative stability, giving the membrane durability under stressful conditions. Abdullaha et al. [6] described a common spinning process with a dope solution of 15 wt.% PVDF in 85–76 wt.% *N*,*N*-dimethylacetamide (DMAc) and 0–9 wt.% commercial TiO_2_ P-25, respectively, and a bore liquid of 80% DI water and 20% DMAc (flow rate 1.5 min/s, spinneret dimensions OD/ID 1.0/0.5 mm). These TiO_2_/PVDF HFs are evaluated in a batch photoreactor hosting a module of 16 bundles immersed in 400 mL of the 1 mol/L MB solution and illuminated by one UV lamp (175 W, 100 mW/cm^2^) placed 10 cm above the glass reactor cell. The efficiency was found to increase with the TiO_2_ content as expected.

**Figure 5:**
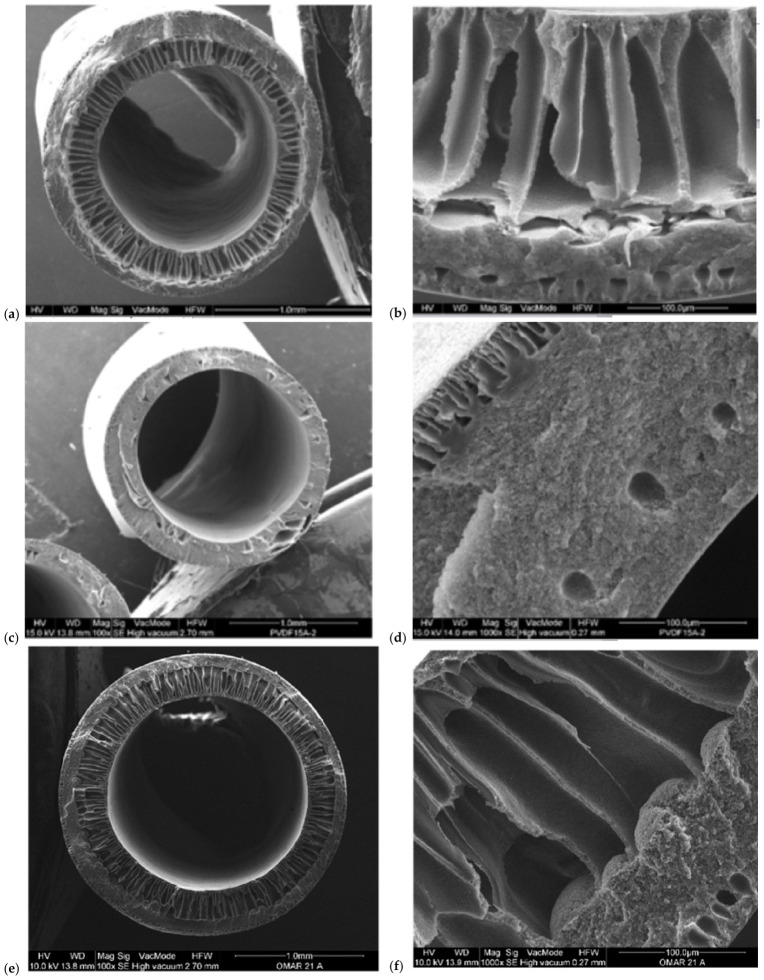

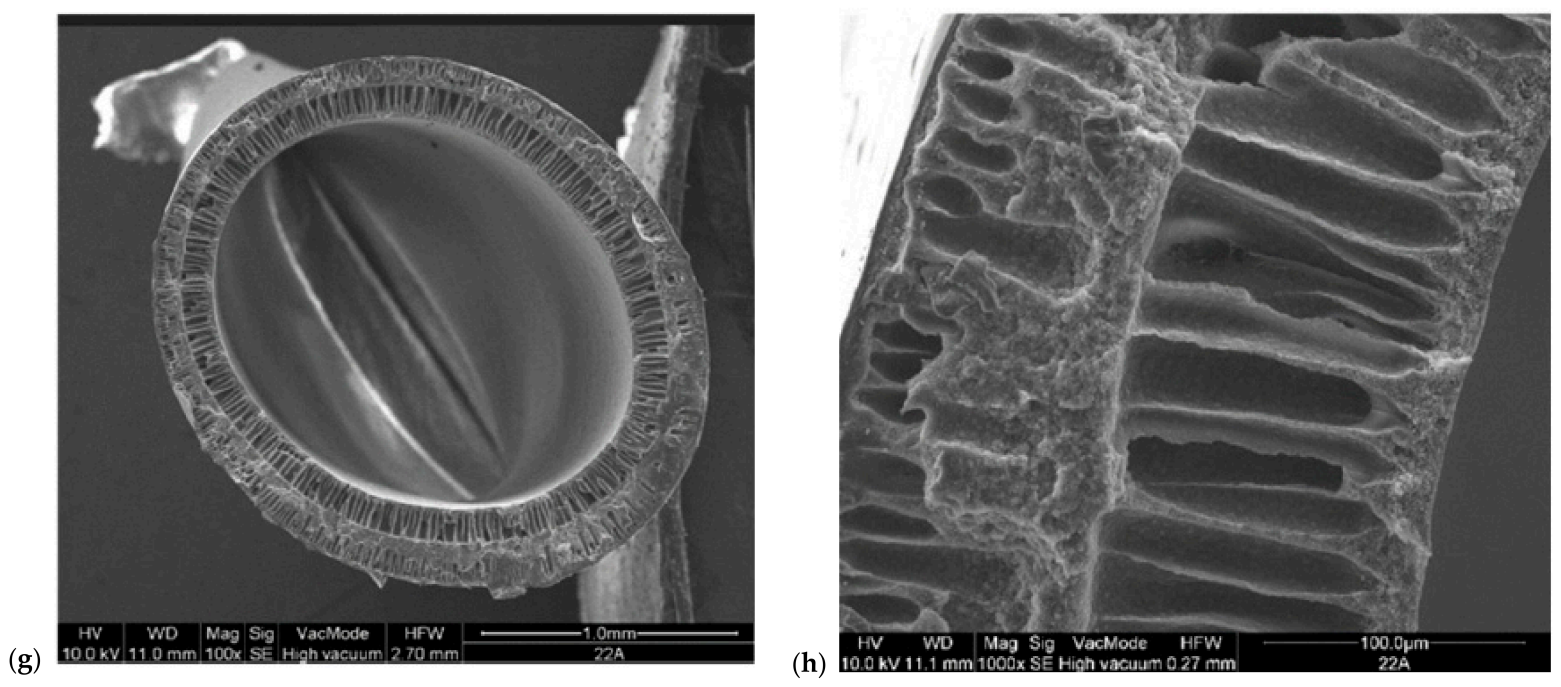
(**a**) 0 wt% TiO_2_, 0 wt% PEG400 (100×); (**b**) 0 wt% TiO_2_, 0 wt% PEG400 (800×); (**c**) 0.5 wt% TiO_2_, 0 wt% PEG400 (100×); (**d**) 0.5 wt% TiO_2_ 0 wt% PEG400 (800×); (**e**) 0 wt% TiO_2_, 10 wt%PEG400 (100×); (**f**) 0.5 wt%TiO_2_ 10 wt% PEG400 (800×); (**g**) 0 wt% TiO_2_, 10 wt%PEG400 (100×); (**h**) 0.5 wt% TiO_2_ 10 wt% PEG 400 (800×) (reproduction from open access [8], no permission needed).

Galiano et al. [8] reports two different routes of preparing the PVDF/TiO_2_ dispersion, combining chemical with mechanical modification of the TiO_2_ NPs, aiming to improve its stability. The two routes’ basic difference was the use of additive PEG400. Firstly, 1 g TiO_2_ (Degussa P25) was chemically functionalized by being added into an anionic surfactant, sodium dodecyl sulphate (SDS) aquatic solution (1.4 g/200 mL DI water), followed by 6 h stirring, centrifugation at 6000 rpm for 10 min, and dried. In the second approach, the functionalized TiO_2_-SDS NPs were subsequently manually grounded to a fine powder using a mortar, and the mechanically modified TiO_2_ was added into NMP solvent under stirring to obtain a stable dispersion. The additives (PVP k17, PEG400) and polymer (PVDF) were slowly added into the heated at 80 °C solvent under stirring and spun with a dope flow rate of 11–12 g/min through an OD/ID 1.6/0.6 mm spinneret (bore fluid: 30% NMP, 13 g/min, 50 °C). The produced HF entered the tap water coagulation through a 24 cm air gap at room temperature, was kept overnight in a sodium hypochlorite (NaClO) 4 g/L solution for PVP removal and soaked for 4 h in a 30 wt.% glycerol aqueous solution to avoid porous structure collapse. The SEM images of Figure 5 show the difference in morphology of the HFMs prepared with and without the addition of TiO_2_ and PEG400 additive and their catalytic contribution to the formation, the shape, and the size of the “finger-like” pores of the HFMs. Tested as filters (under flow conditions), the HFMs exhibited water permeability values between 51 and 81 L/m^2^hbar. Their photocatalytic activeness was tested under 0.5 bar transmembrane pressure and the flow velocity at 0.06 m/s. Three HFMs of 20 cm (total surface area 0.0036 m^2^) were tested for the decomposition of 250 mL 10 μm/L MB, under 2 UV-A light (18 W, λ_max_ at 365 nm) at a distance of 6 cm from the module, with the estimated light intensity reaching the membrane surface being 2.7 mW/cm. The rejection was found to be of 97%.

A recent work [30] used PVDF in DMAc with the addition of 2% *w*/*w* of TiO_2_ nanoparticles and 5% *w*/*w* of PVP to form the dope dry-jet wet spinning solution. A bundle of 10 of the occurring HFMs, of 20 cm each, with a total effective membrane area of 0.66 ± 0.04 m^2^, were tested against carbaryl pesticide solutions (0.3 to 20 mg/L) in flow conditions. The PVDF-TiO_2_ HFMs, under a 60 W UV, exhibited 60% carbaryl removal efficiency, followed by a reduction to their mechanical properties, as indicated by the vanishing of the α-crystalline phase in PVDF, due to UV exposure.

### 2.2. Dual Layer Coextrusion

PVDF has also been used for the fabrication of dual-layer (DL) photocatalytic HFs via a single-step coextrusion of two different dope solutions through a triple-orifice spinneret. In most cases, the inner layer consisted of PVDF in DMAc (*N*,*N*-dimethylacetamide) solvent, while the outer layer was a mixture of PVDF, TiO_2_, and DMAc in various concentrations.

Kamaludin et al. [53] used TiO_2_ P25 Evonik nanoparticles in a dry/wet spinning method (10 cm air gap) involving outer dope composition of wt.% 15/3/82 PVDF/TiO_2_/DMAc and inner dope composition of wt.% 18/5/77 PVDF/PEG/DMAc with spinneret dimensions of 0.8/1.2/2.6/3.0/3.5. Membrane permeance is known to increase analogically with the length of the finger-like structures in the produced membrane morphology, which, subsequently, increases with the addition of additives in the dope solutions. Polyethylene glycol (PEG) is one of them, which, when of an elevated molecular weight, can promote the formation of a porous structure. The outer dope’s flow rate was of 2 mL/min, while the inner dope’s and the bore fluid’s (distilled water) flow rate were 8 mL/min. The spun fibers were immersed for 1 h in a 50:50 wt.% mixture of ethanol/water, followed by 100% ethanol for another 1 h, to prevent pore collapse. DLHFs were also prepared by triple-orifice spinneret extruding of two different dope solutions simultaneously, the inner of which consisted of 18 wt.% PVDF and 82 wt.% of DMAc solvent, while the outer layer was a mixture of 15 wt.% PVDF, 3 wt.% TiO_2_ and 82 wt.% DMAc [29,54]. The DLHFs were tested photocatalytically against eight target pharmaceuticals present in groundwater and secondary wastewater effluent. According to the findings, Metoprolol and Trimethoprim exhibited the highest kinetic constants (k > 0.08 min^−1^), higher than those obtained with un-mobilized TiO_2_ Degussa P25 (k < 0.05 min^−1^), while for carbamazepine, the differences seemed lower (k > 0.06 min^−1^ vs. k around 0.04 min^−1^) [29]. Determination of electrical energy per order of magnitude of transformation (EEO) also took place, confirming the lower energy demands for the transformation of the selected pharmaceuticals in secondary effluent by the TiO_2_ DLHFs (33–58 kWh/m^3^ compared to 49–79 kWh/m^3^ when applying only photolysis).

Dzinun et al. reported a solution preparation involving dispersion of TiO_2_ nanoparticles (average particle size 16 nm, surface area 72 m^2^/g) in DMAc and 24 h stirring, followed by gradual addition of PVDF and ultrasonication [26,54]. The same team also studied the performance of PVDF DLHFMs after the addition of 5 wt.% of PEG 6000 to the inner dope solution, and 3 wt.% of TiO_2_ nanoparticles to the outer dope solution [55], along with portion TiO_2_/PVDF variation from 0 to 1 [37,54]. The above-described DLHFs were tested photocatalytically against nonylphenol (NP), an endocrine disruptive compound, in batch [26] and flow [37] conditions. A module holding 20 HFs of 23.5 cm each (248 cm^2^ total area) was immersed in the 10 ppm NP feed solution (30 mg of in 3 L H_2_O: acetonitrile 9:1 mixture) and irradiated by a UVA lamp of 8 W (365 nm, light intensity 0.33 mW/cm^2^) for 4 h. As expected, the sample with the maximum TiO_2_ content proved the best performing one, decomposing NP within 150 min, with a degradation rate of K_app_ = 0.0173 min^−1^ [26]. The same DLHFs have also been used under flow conditions, an application which turns them into DLHFMs. Specifically, the antifouling properties when being penetrated by the NP solution were checked. Under UV irradiation, an increase in the NP flux was observed, which was strongly related to the distribution of TiO_2_ nanoparticles at the external layer of the DLHF membranes. The best performing sample was that of 0.2 TiO_2_/PVDF ratios, with an NP flux value of 25 L/m^2^h. Lamps of both 8 W and 36 W were used, to ensure the activation of the superhydrophilicity of TiO_2_ when irradiated with higher light intensity (18.2 mW/cm^2^).

The most recent publication on photocatalytic DLHFs [40] reports the use of a TiO_2_-WO_3_@GO photocatalyst in a coextrusion/phase inversion fabrication method. The composition of the inner layer dope was PVDF/PEG/DMAc 18/3/79 wt.% and that of the outer layer was DMAc and TiO_2_-WO_3_@GO photocatalyst powder with varying (0,1,3,5) wt.%. These TiO_2_-WO_3_@GO/PVDFs were irradiated for 6 h using one 100 W LED (visible light) bulb. A bundle of 20 HFMs of 10 cm in length, potted in a PVC module, participated, with oilfield-produced water (OPW) being the foulant. The best TOC rejection (98.62%) was exhibited by the 3 wt.% loaded membrane after 6 h of operation. The absorptive capacity of TiO_2_ in the visible range proved to be improved by the electron storage of WO_3_ within the system and promoted by the electron–hole transfer of the GO. Therefore, the TiO_2_ band gap value was 2.32 eV to 2.16 eV. The HFMs’ initial water permeability and oilfield-produced water (OPW) flux were 99.51 L/m^2^h and 76.54 L/m^2^h, respectively, while after five cycles of operation, their pure water permeability and OPW flux were 94.02 L/m^2^h and 69.92 L/m^2^h, respectively. The corresponding measured values for the nonphotocatalytic (0 wt.% TiO_2_-WO_3_@GO) membranes were only 24.86 L/m^2^h and 12.58 L/m^2^h, proving the presence of photoinduced hydrophilicity.

**Figure 6:**
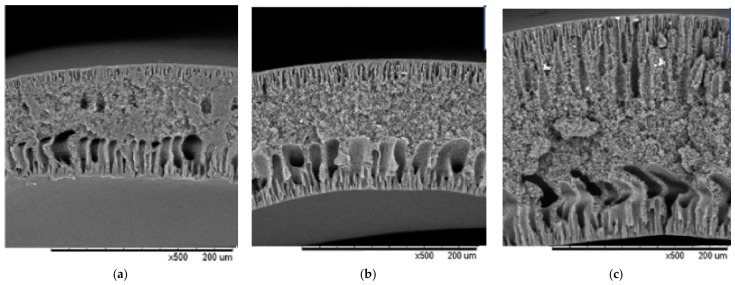
SEM image of 0.25 Cu_2_O/PVDF DLHF membrane with different outer dope flowrates: (**a**) 3 mL/min, (**b**) 6 mL/min, (**c**) 9 mL/min (reproduction from open access [20]).

Another dry–wet coextrusion case was that of forming Cu_2_O/PVDF DLHFs [20]. This study carried out an investigation regarding the best Cu_2_O loading and the best outer dope extrusion flow rate (3, 6, or 9 mL/min) when inner dope and bore fluid flowrates were kept constant at 26 and 8 rpm, respectively (Figure 6). The dope was extruded through a triple-orifice spinneret, through an air gap of 100 mm. The post-fabrication membrane treatment process involved soaking of the HFs in a water bath for 24 h for residual solvent removal, 1 h immersion in 50:50 wt% ethanol, and 1 h pure ethanol, to prevent the membrane from shrinking. The embedding Cu_2_O, with a narrow band gap of 2.2 eV, PVDF DLHFs, were tested for batch (not flow) photocatalytic degradation under visible light and successfully eliminated 75% of the 10 ppm BPA in 360 min with no observed Cu leach.

The same triple-orifice equipment dry/wet procedure was followed by Zakria et al., employing PVDF and copper (II) oxide and vanadium tetrasulfide (CuO/VS_4_) as visible light active photocatalyst [21]. The inner layer dope consisted of PVDF, PEG6000 and DMAc (15, 2, 83 wt%) stirred at 60 °C, while the outer layer dope of PVDF, DMAc and CuO–VS_4_ at three different concentrations wt% (a) 15, 1, 84, (b) 15, 3.75, 81.25 and (c) 15, 7.5, 77.5. The spinning parameters were 10 cm air gap, 8 mL/min inner dope and bore liquid flow rate, with 6 mL/min outer dope flow rate. The coagulation/phase inversion baths changed from water to 50% ethanol (1 day) to absolute ethanol (1 h). The prepared HFMs were evaluated under batch and flow conditions for the photodegradation of 1 mg/L BPA under visible light. The membrane with 3.75 wt% photocatalyst performed 73.19% BPA photodegradation and rejection of BPA after 4 h.

Zakria et al. [25] also synthesized a PVDF/MoO_3_/ZnO/GO DLHF, which exhibited excellent photocatalytic degradation performance against endocrine disruptive compounds (EDCs). The 0.5 PVDF/MoO_3_/ZnO/GO DLHF reached a 74.02% regeneration efficiency after three consecutive cycles. The same process was obtained recently [31] for the fabrication of PVDF/3Al_2_O_3_*2SiO_2_/TiO_2_ DLHFs, which by employing 4 g catalyst in batch conditions, removed 44.92% of COD and 52.40% of color loadings under a 6 W UV lamp. It also showed a 54.5% rejection of a POME solution in flow conditions.

Table 1 presents, comparatively, the various structural/operational parameters encountered within the mixed matrix spinning fabricated photocatalytic HFs/HFMs. As seen, a direct comparison between them in terms of efficiency is not actually achievable, as there is not a uniform protocol or standard way of evaluating the efficiency of a catalyst. The experiments described differ in terms of the pollutant used, the catalyst quantity, and the illumination parameter, as well as whether the actual application is a batch or a flow one. Additionally, some crucial parameters, such as the surface area or the pore size of the HFs/HFMs, are not always provided in the articles.

## 3. External Coating of HFs with Photocatalyst

### 3.1. Spinning-Derived HFs/HFMs

**Figure 7:**
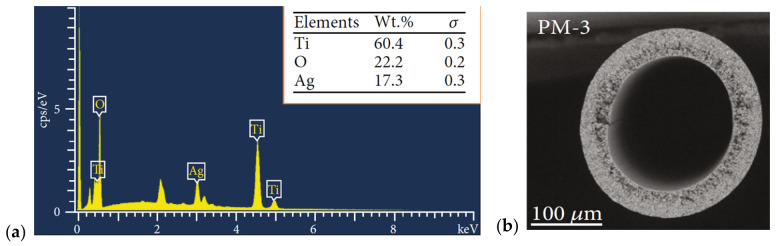
(**a**) EDX analysis of P-4 (1.4 g Ag@TiO_2_). (**b**) SEM images of Ag@TiO_2_ HF membrane PM-3 (IT = 1:3 min) (reproduced from open access Ref. [23]).

Shareef et al. [22,23] reported the external decoration of polysulfone (PSf) HFMs with the Ag@TiO_2_ photocatalyst via the dip-coating technique (Figure 7). The initial spinning dope solution was prepared using the low-cost kaolin powder (Al_2_Si_2_O_5_(OH)_4_) as a source of Al_2_O_3_. Kaolin was immersed into the blend of PSf and NMP at the ratio of 6:1:8, mixed for 2 h, and extruded through an orifice spinneret at a rate of 9 mL/min using water as the bore liquid. Τhe fibers produced were soaked for 24 h in water for NMP to remove before annealing at 1250 °C for 24 h. The 15 cm long HFMs were dip-coated in a solution of 0.6 g Ag@TiO_2_ nanoparticles in 400 mL of DI water followed by sonication for 1 h. Different immersion intervals (0.5, 1.0, 1.3, 2 min) resulted in different HFMs after sintering at 500 °C for 2 h (elevation 2 °C/min). The band gap of the Ag/TiO_2_ photocatalysts was determined at 2.9 eV, while the corresponding value for the Ag/TiO_2_-coated membrane was 2.5 eV. The water permeability of the HFMs was found to decrease with the immersion duration into the Ag/TiO_2_ nanoparticle solution. Specifically, from 625.76 L/m^2^∙h for the TM-0 it dropped down to 75.78 L/m^2^∙h for TM-120, a finding attributed to the nanoparticles’ blocking of the membrane pore. Despite their water permeability testing, the HFMs were further tested photocatalytically under batch conditions. HFs of 8 cm length achieved an 88% BPA degradation (100 mL, 10 mg/L) within 270 min under a 100 W Xe lamp (visible light).

CuO/CeO_2_ photocatalyst coated externally asymmetric Al_2_O_3_ HFMs were prepared through the phase-inversion-based spinning technique [38], by loading Al_2_O_3_ of two different particle sizes (20 and 10 mm) in a N-methyl-2-pyrrolidone (NMP) solution, followed by the addition of a polymer binder at two alumina/PES ratios (10:1 and 6:1). The suspension was spun (spinneret orifice OD/ID (mm) 3.0/2.8, air gap 5–25 cm) to form fibers (OD 1.1–2.0 mm and ID 0.7–1.2 mm) immersed in water for 24 h to complete the phase inversion, straightened, dried, cut into 35 cm and sintered at 1400 °C (temperature ramp 3 °C/min to 400 °C, held for 1 h, then ramp of 4 °C/min to 800 °C, for 2 h, rate 5 °C/min to 1400 °C). Immersion of the fibers into the 1–3 wt.% CuO/CeO_2_ photocatalyst solution took place after preventing the catalyst solution from entering the alumina lumen by wrapping the ends of the HFMs with polytetrafluoroethylene (PTFE) tape. After drying at 115 °C for 2 h, the calcination process was carried out at 400 °C for 1 h. These membranes were tested for antifouling and self-cleaning after being exposed to UV irradiation, under 2 bars transmembrane pressure. The pristine HFM’s water permeability was 118.5 L/m^2^hbar and reduced to 107.4 L/m^2^hbar at the introduction of 10% BSA solution in the feed stream. The corresponding values for the CuO/CeO_2_ HFMs were 56.5 L/m^2^hbar and 19.0 L/m^2^hbar. The latter value increased to 30.5 L/m^2^hbar after being UV-irradiated.

PVDF HFMs have been used as a substrate for TiO_2_ nanoparticles’ external coating through a chemical binding on the polymer [42]. For this work, dopamine was considered an excellent linkage due to it being multifunctional, processing both amino and hydroxyl groups. The dope solution for the spinning (1000 μm OD, 600 μm ID) involved dissolving 9.6 g PVDF and 3 g PVP in 47.4 g NMP under heating and stirring. The extruded PVDF HFs were immersed into 0.2 wt.% dopamine buffer solution (Tris-HCl, pH 8.5), soaked in 0.05 wt.% NTN buffer solution (Tris-HCl, pH 8.5) at 35 °C for 2 h, and rinsed with ethanol and DI for the removal of any loosely bound dopamine.

PVDF HFMs (no more manufacturing information provided) have been reported to be modified with sandwich-structured WO_3_/g-C_3_N_4_/CNTs photocatalyst via vacuum filtration (P = −0.06 MPa, J_0_ = 110 L/h/m^2^, flow rate 0.40 L/h) [34]. Cut in pieces of 5 cm in length, with a total area of 3.67 × 10^−3^ m^2^, the HFMs were fixed into a curtain-like module. CNTs were first pumped, to create a membrane pore blocking phenomenon, followed by the adsorption of the pollutant molecules (MB). Afterwards, a filtration of the WO_3_/g-C_3_N_4_ took place, to form an overall sandwich-like structure with the photocatalyst loaded on the outermost layer, to be fully in contact with light. Visible light irradiation was provided by a 350 W xenon lamp. When operating under flow conditions (P = −0.06 MPa), the MB removal rate of the membrane module remained at 89.47% after 4 h of operation and the flux of the membrane module decreased slightly.

Wan et al. [41] also reported the chemical bonding of N-doped TiO_2_ on the top of PSf HFs, prepared by the common spinning process. A 3-(3,4-dihydroxyphenyl) lalanine (LDOPA) layer was then attached onto their surface via self-polymerization as they were soaked in a 0.2 wt.% LDOPA (Tris (hydroxymethyl)-aminomethane hydrochloride) buffer solution of pH 8.5 for 6 h, 12 h, 18 h, 24 h, followed by rinsing with ethanol and DI water three times to remove any loosely bound LDOPA. The N–TiO_2_–NH_2_ (NTN) introduction to the LDOPA layer via chemical bonding occurred in a 0.05 wt.% NTN Tris-HCl pH 8.5 buffer solution at 35 °C for 2 h. These HFMs proved to have self-cleaning antifouling properties under visible light irradiation. Despite being long-term instable, the modified HFM proved able to recover completely to its initial pure water permeate flux when irradiated with visible light, although being previously exposed to the model foulant humic acid (20 mg/L, pH = 7).

PES was the precursor polymer also in the fabrication of the only photocatalytic HFMs utilized in gas application [39]. Ceramic HFMs were prepared using an extrusion suspension of 60% α-Al_2_O_3_ powder (0.5 μm particle size) and 40% additives (PES, PVP, NMP). The extrusion occurred through an iron nozzle at 3 bar pressure, with an air gap of 10 cm. The formation of the fiber followed sintering at 1300 °C for 3 h. Nitrogen-dopped (N-TiO_2_) and undoped TiO_2_ were applied on top of the Al_2_O_3_-based HFMs by wash-coating (1 h dip) based on tetraethyl orthosilicate (TEOS) solution as a silica-based binder. Heterogeneous complete (100%) photocatalytic degradation of gas pollutant NH_3_ took place in a special custom-made flow gas reactor, utilizing 42 Al_2_O_3_-based HFMs exposed to UV and visible light, within 15 min.

Alias et al. [39] describes a hybrid process, combining the spinning/sintering method with the electrospinning HF manufacturing, producing graphitic carbon nitride (g-C_3_N_4_) photocatalyst nanofiber-coated Al_2_O_3_ HFs. The Al_2_O_3_ HFMs are prepared through a typical spinning/phase inversion method using three different kinds of alumina powders: (a) α-Al_2_O_3_ (99% metal basis, 1 μm average particle size, 6–8 m^2^/g surface area), (b) α- and γ-Al_2_O_3_ (99.5% metal basis, 0.5 μm average particle size, of 32–40 m^2^/g surface area), and (c) α- and γ-Al_2_O_3_ (99.8% metal basis, 0.01 μm average particle size and 100 m^2^/g surface area). The three Al_2_O_3_ powders were mixed at a ratio of 1:2:7, and 106 g of the mixture powder was added into 73.74 g of NMP solution containing 2.6 g of the commercial additive Arlacel P135, before 17.66 g of PES was added. The as-prepared suspension was extruded to form PES/Al_2_O_3_ HFMs, later sintered at 1400 °C. At the second step, a solution containing 7.2 wt.% of polyacrylonitrile (PAN) and 0.8 wt.% of g-C_3_N_4_ was electrospun on the ceramic Al_2_O_3_ HFMs. The 7 cm in length Al_2_O_3_ was fixed on a holder placed 18 cm away from the needle, and directed perpendicular to it, while rotating at 6 rpm. The solution feed rate was 1 mL/h and the acceleration voltage was 15 kV, which homogeneously spun the nanofibers on the membrane, forming a mesh. The membrane was tested in a cross-flow filtration mode for antifouling and self-cleaning properties, under UV irradiation (30 W lamp, peak at 312 nm). The NF-g-C_3_N_4_/Al_2_O_3_ membrane exhibited 99% oil rejection, pure water permeability of 816 L/m^2^h and a high OPW permeability of 640 L/m^2^h. After three cycles of operation, the membrane maintained a high permeate flux of 577 L/m^2^h and a good oil rejection (97%) as a result of the degradation of the captured oil foulants promoted by the g-C_3_N_4_ nanofiber coating after 180 min of irradiation.

Ceramic HFMs (ID: 1.3 mm, OD: 2.0 mm, length: 10 cm, average pore size: 1.21 μm, porosity: 59.09%), derived from a spinning procedure using a dope mixture of Al_2_O_3_/NMP/PES/PVP in the ratio of 100:56:10.8:0.8, were externally decorated with S-doped titania nanotubes [43]. The catalyst immobilization took place via filtration of a S-TiNTs suspension (100 mL, 0.2 g/L) through the HFM by applying a 0.088 MPa vacuum; followed by sintering at 500 °C for 2 h. The prepared fibers were tested photocatalytically in batch conditions under simulated sunlight (xenon lamp system, λ > 320 nm, 220 mW/cm^2^) positioned at a 15 cm distance, degrading 99.89% of an MO solution (10 mg/L, 50 mL, pH = 7) in 180 min. Additionally, after being statically illuminated for 1 h, the HFMs achieved flux recovery rates of 85.8% and 57.6% for separating engine oil emulsions and OPW, which indicates high self-cleaning, antifouling performance.

### 3.2. Commercial HFs

PES and blended polyvinyl-chloride/polyacrylonitrile (PVC-PAN) commercial HFMs have been used as photocatalyst immobilization mediums, due to their good chemical and mechanical stability and high specific surface [9]. Attempts of external decorating with TiO_2_ took place by spray coating (0.1% *w*/*v*) from a distance of 10 cm, while the fibers were placed horizontally and rotated at 20 rpm. Other attempts involved vacuum coating through filtration, with the suspension of 0.1% *w*/*v* TiO_2_ fed to the shell side of the HFMs, whereas the permeate was collected at the lumen. Vacuum was applied to the lumen side to ensure the continuous permeation of the liquid through the membrane, enforcing attachment of nanoparticles onto its porous matrix. In both spray and vacuum coating routes, detachment was observed as the nanoparticles were immobilized physically. The aim of maximizing the photocatalyst loading while minimizing the permeability loss of the coated membrane was achieved through the sol–gel dip-coating method, in solutions of 0.1 wt.% and 0.17 wt.% TiO_2_ and 10 s dipping time, with a downward/upward velocity of 2 mm/s. The HFMs were evaluated for the photocatalytic activity against MB and Chlorhexidine Digluconate (CHD) pharmaceutical under simulated solar light of 1500 W. The membrane glass module (12 cm long, 1.2 cm diameter) used accommodated four HFMs (10 cm long, 7.22 cm^2^ available surface area) and was tested in the cross-flow mode, with the transmembrane pressure varying from 40 to 60 kPa by partially closing the retentate valve. For the operation of the coated UF membranes, the “outside–inside” configuration was chosen, as the skin layer was on the outer side of the fiber, achieving a degradation of MB and CHD at 30% and 40%, respectively.

Table 2 summarizes the articles describing the external coating of photocatalyst onto spinning-derived or commercial HFs/HFMs and their structural/operational parameters. As seen, most of them choose the dip-coating, as spray and vacuum deposition seem to apply the catalyst layer in a nonreproducible and long-term stable manner [9].

### 3.3. Electrospinning Method

The electrospinning technique produces ultra-fine 1D fabric materials by viscous solutions or melts. Polymeric, inorganic or hybrid composites with controlled compositions can be tuned in multistructure configurations with superior properties and functionalities, appropriate for photocatalytic applications.

**Figure 8:**
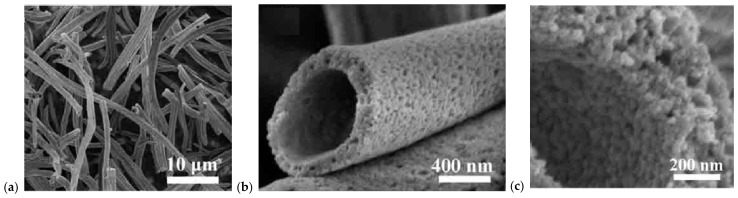
Typical SEM image of the calcinated HFs under (**a**) low magnification, (**b**,**c**) higher magnifications (reproduced from open access Ref. [16]).

Hou et al. report the fabrication of mesoporous TiO_2_ HFs (Figure 8a) via a single capillary electrospinning method, according to which foaming agents are used to create the walls’ mesoporous structures [16]. The electrospinning route used 0.6 g Polyvinylpyrrolidone (PVP, average MW ≈ 130,000), 0.5 g diisopropyl azodiformate (DIPA), and 3.0 g Ti(OBu)_4_ (TBOT) as Ti precursor. After dilution in 7 mL absolute ethyl alcohol and vigorously stirring for 2 h to form microemulsions, 0.5 g of cetrimonium bromide (CTAB) and 2 mL paraffin oil were added, and the solution was transferred into a stainless-steel nozzle with a 0.2 mm diameter. The nozzle’s tip (anode) was placed in front of a metal cathode (collector) at ae distance of 20 cm. A flow rate of 1 mL/h and an electrical potential of 18 kV a were applied. The as-spun fibers were heated up to 500 °C (rate 1 °C/min). The formation of the hollow interior (Figure 8b) can be assigned to the trapped paraffin oil, which, once subjected to 500 °C, is decomposed into CO_2_ and gas H_2_O. The HF’s mesoporous walls (Figure 8c), on the other hand, should be mainly ascribed to the thermal decomposition of foaming agents (DIPA) into CO_2_, NO_2_ and H_2_O. The obtained TiO_2_ HFs (94.6% anatase/5.4% rutile) degraded at 99.5% 10 mg/L Rhodamine B (RhB) in 60 min and achieved 499.1 μmol/gh H_2_ production under the irradiation of a 300 W xenon lamp (λ > 320 nm).

Zhan et al. [1] introduced the fabrication of core–shell TiO_2_ HFs 30 cm long with a combined two-capillary spinneret electrospinning technique and co-electrospinning apparatus. The triblock copolymer luronic P123, (H(C_2_H_5_O)_20_(C_3_H_7O_)_70_(C_2_H_5_O)_20_OH) was used for pore directing, a mixture of which, with aged inorganic precursor Ti(OBu)_4_ solution, was added into the outer spinneret tube. An immiscible liquid (machine oil) was poured into the inner capillary. N_2_ pressurized the two fluids and drove them to the spinneret’s tip, which was supplied with high voltage (25 kV). The distance between spinneret and collector was 30 cm, and the collected fibers were soaked in cyclohexane for 24 h for the thorough extraction of the machine oil. The adjustment of the oil and sol pressures to HFs with tunable inner diameters (0.1–4 μm) and wall thicknesses of 60–500 nm, along with the consequent phase inversion, led to inorganic HFs with mesoporous walls 6.7 nm in diameter. The HFs exhibited higher photocatalytic activities than commercial TiO_2_ P25 toward the degradation of MB (100 mL, 2 mg/L), which, under a 125 W UV mercury lamp (λ = 320–400 nm, λ_max_ = 365 nm), almost disappeared after 40 min of irradiation.

A case of electrospun HFs employing a photocatalyst beyond the usual TiO_2_ is that of ZnO-SnO_2_-Zn_2_SnO_4_ and Zn_2_SnO_4_ [10]. The precursor solution resulted after a simultaneous mixing of 1.42 g tin acetate, 1.756 g zinc acetate and 1.5 g of methyl methacrylate (PMMA) in 15.8 mL of *N*,*N*-dimethylformamide (DMF). This blend was injected through a stainless-steel needle (25 gauge, orifice diameter = 250 μm) connected to a 17.25 KV supplier, with a continuous injection rate of 1.2 mL/h. The rolling drum was located 15 cm below the needle, and Zn_2_SnO_4_ HF was formed afterwards by calcining at 1000 °C. The HFs were tested photolytically against MB, CR, phenol and BPA solutions of 10^−5^ M in the presence of 100 mg photocatalysts. They degraded 64% and 49% of Phenol and Bisphenol A, respectively.

Nguyen et al. [11] prepared dense TiO_2_ and macroporous TiO_2_ hollow fibers using PVP as a stabilizer and titanium diisopropoxide bis (acetylacetonate) (TDIP, 75% in isopropanol) as Ti precursor material. Here, 1 g PVP was added to 7 mL ethanol at room temperature stirring for 2 h, followed by 2 mL TDIP and 0.67 mL 0.01 N HCl for hydrolysis. The spinning solution and the core–shell oil was fed through two syringe nozzle pumps with flow rates of 15 and 3 μL/min, respectively. Then, 12 kV was applied and the distance between the nozzle and the collector was fixed as 10 cm. For the formation of the macroporous fibers, PS template nanospheres (190–470 nm) were added to the outer spinning solution, which were removed by the final calcination at 500 °C. The inner dope oil was also removed using hexane to form the hollow structure. The best photocatalytically performing hollow titania fiber had a rate constant of 0.0772 min^−1^.

**Figure 9:**
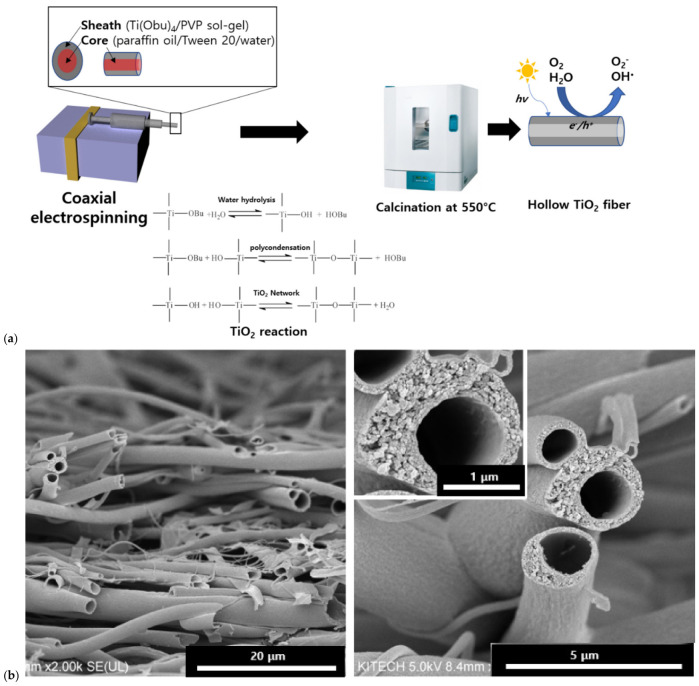
(**a**) Fabrication method of hollow TiO_2_ fibers via coaxial electrospinning and their reactions for the hydrolysis process with titanium butoxide as a precursor. (**b**) SEM images of the TiO_2_ HFs at various magnifications (reproduction from open access reference [47]; no permission needed).

A coaxial electrospinning experimental setup was used by J. Kim [47] for the formation of TiO_2_ HFs, with diameters of OD/ID 3.0/2.5 mm and 0.6/0.37 mm, respectively. For the blend formation, a 40 wt.% Ti(OBu)_4_ solution was gradually added to a PVP in ethanol and acetic acid (1:8:1 *w*/*w*/*w*) solution at room temperature under 4 h of stirring. Paraffin oil emulsion was simultaneously fed into the inner capillary with core flow rates of 0–3 mL/h (Figure 9a), while the outer flow rate was fixed at 8 mL/h. The voltage applied was 25–30 kV and the work distance between the spinneret and collector was 15–20 cm. The collected fibers (Figure 9b) were calcinated at 550 °C for 1 h with a heating rate of 1 °C/min to remove the polymer and the paraffin oil emulsion. The best performing sample exhibited a 66.2% NO removal from a 1 ppm and 3 L/min flow rate NO stream with a relative humidity (RH) of 50% at 25 °C.

The same technique was used in a very recent article [45] for the development of Fe-doped TiO_2_ HFs, to be used as a substrate for the growth of UIO-66 MOFs. The composite material was tested for synergic adsorption and photocatalytic degradation of acetaldehyde. The samples were irradiated vertically by a UV (405 nm) and a visible LED light (550 nm) lamp with densities of 1.10 mW/cm^2^ and 2.59 mW/cm^2^, respectively. Based on these values, photon fluxes were calculated to UV ≈ 2.24 × 10^15^ and visible ≈ 7.17× 10^15^ photons/cm^2^/s. Under 30–40% relative humidity (RH) the removal of acetaldehyde was 69.8% under UV, 37.9% under visible, and 73.3% under combined UV/Vis irradiation.

Table 3 presents the comparative structural and operational parameters of the electrospinning-derived photocatalytic HFs encountered in the literature.

### 3.4. Template Method

The templating method is commonly used to generate hollow structural materials, with the template materials forming the inner layer, while precursor materials are coated onto them. Repeating the coating procedure can form a core–shell structure, while the hollow structure occurs by subsequent removal of the core part. Being of low cost and widespread in nature, biotemplates like cellulose, in the form of cotton fibers [2] and kapok [3,4,28], have been reported, all using Ti(OBu)_4_ as Ti precursor.

**Figure 10:**
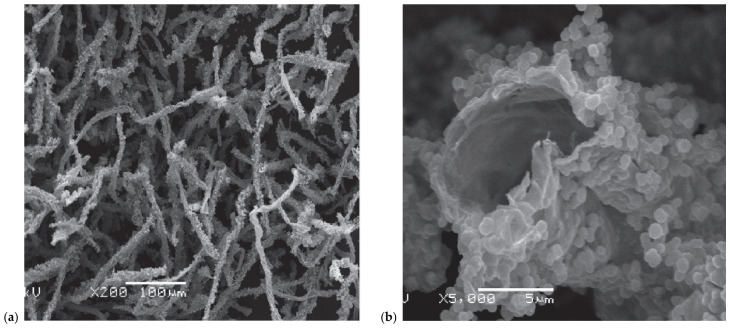
SEM micrographs of the sample 600 °C (maximum anatase) at magnitudes of (**a**) 100× and (**b**) 5000× (reprinted with permission from Ref. [2]; copyright year 2012, American Chemical Society).

Cotton was the first template used for the preparation of photocatalytic HFs [2]. The precursor Tetrabutyl titanate (TBT) solution was prepared by its addition to 75 mL of anhydrous alcohol, in which the dry cotton fibers were immersed. A calcination step (450–700 °C, 2 h, air) followed the template removal for the transformation of Ti^4+^ to TiO_2_. Their evaluation against MB took place under a xenon lamp of 800 W. The sample calcinated at 600 °C outperformed the rest, as it possessed the maximum anatase content (Figure 10).

The same process was followed when replacing cotton fibers with kapok and using titanium butoxide (TiBut) as Ti source. [28]. These THFs photocatalytically degraded Herbicide Paraquat (GMX) (100 mL,10 ppm) under 480 min UV (6 W, λ = 365 nm). The highest degradation reaction rate (k = 1.39 × 10^−3^ min^−1^) was determined for THFs calcined at 450 °C, and gradually decreased as the calcination temperature increased.

Following the same procedure, the TiBut Ti source was enriched with Fe(NO_3_)_3_·9H_2_O at concentrations up to 5% for the formation of Fe-dopped TiO_2_ [3]. In that case, Wongcharoen et al. [3] reported an interesting decrease in the photocatalytic activity of the HFs. Under 20 W LED irradiation for 6 h, MB (50 mL, 5 ppm) was degraded by 67%, 66%, 55%, and 49% for THF, with 1–Fe-THF, 3–Fe-THF, and 5–Fe-THF, respectively. Although Fe molecules were expected to narrow the band gap and bring activity to the visible region, high levels of Fe dopants served as a recombination center for holes and electrons, instead of electron scavengers.

Although not describing their fabrication process, Lu et al. [41] used calcium alginate fibers as a template to form In_2_O_3_/ZnIn_2_S_4_ HFs. Here, 10 g calcium alginate fibers were added to a (CH3COO)_3_In solution; after ion-exchange/annealing, In_2_O_3_ hollow nanotubes were obtained, to be afterwards decorated externally with ZnIn_2_S_4_. For the photocatalytic H_2_ evolution reaction, a 300 W xenon lamp with a 420 nm cutoff filter and a power intensity of 173 mW/cm^2^ was used. After optimizing the loading of ZnIn_2_S_4_, the In_2_O_3_/ZnIn_2_S_4_ heterojunction achieved a photocatalytic HER rate of 2.18 mmol/g/h under visible light irradiation.

**Figure 11:**
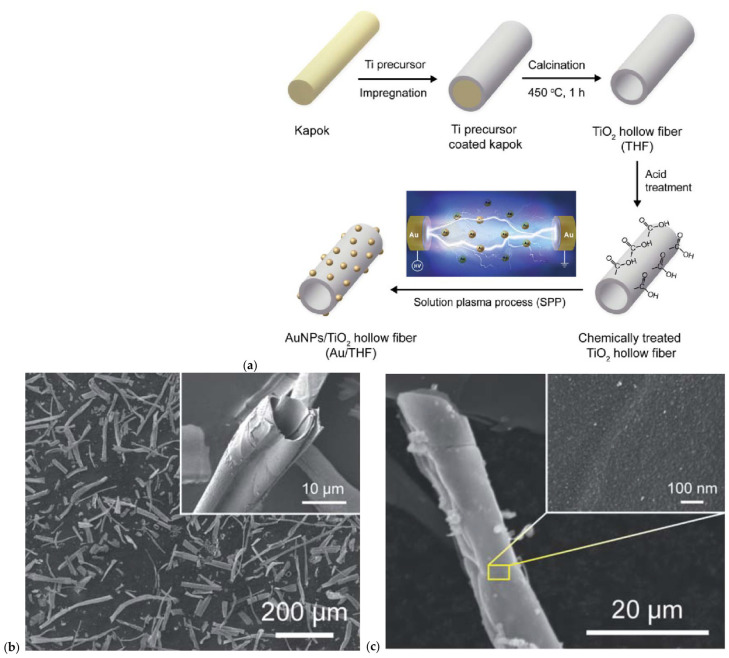
(**a**) Schematic diagram depicting the overall synthesis of by template method and SPP. (**b**,**c**) SEM images of the Au/THFs in different magnifications (reproduction from reference [4]; copyright year 2022, Royal Society of Chemistry).

Panomsuwan et al. [4] decorated the (as above prepared) THFs with Au nanoparticles after acid activation and plasma solution treatment (Figure 11a); however, their diameters (Figure 9a) became not easily adjustable. The Au-THFs were tested photocatalytically under a 50 W Vis–LED lamp, situated at a distance of 10 cm, against MB (100 mL, 10 mg/L). Within 8 h, at the dosage of 1 g/L, THFs and Au/THFs degraded 46% and 88% of the MB load, respectively. The overall Au/THFs’ photocatalytic activity was found to be double that of the bare THFs, and the photodegradation reaction was almost three times faster.

Table 4 summarizes the articles describing template fabrication of photocatalytic HFs and their structural/operational parameters. Although the technique is high-energy demanding for the sintering removal of the template, in all cases, the samples occurring from the lowest calcination temperature (450 °C) appear to be the best performing.

### 3.5. Combined Template/Electrospinning Route

**Figure 12:**
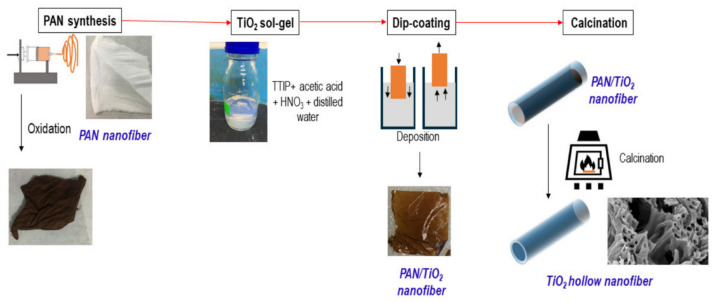
Schematic diagram of TiO_2_ hollow nanofibers’ development via template synthesis (reproduction from open access ref. [18]).

**Figure 13:**
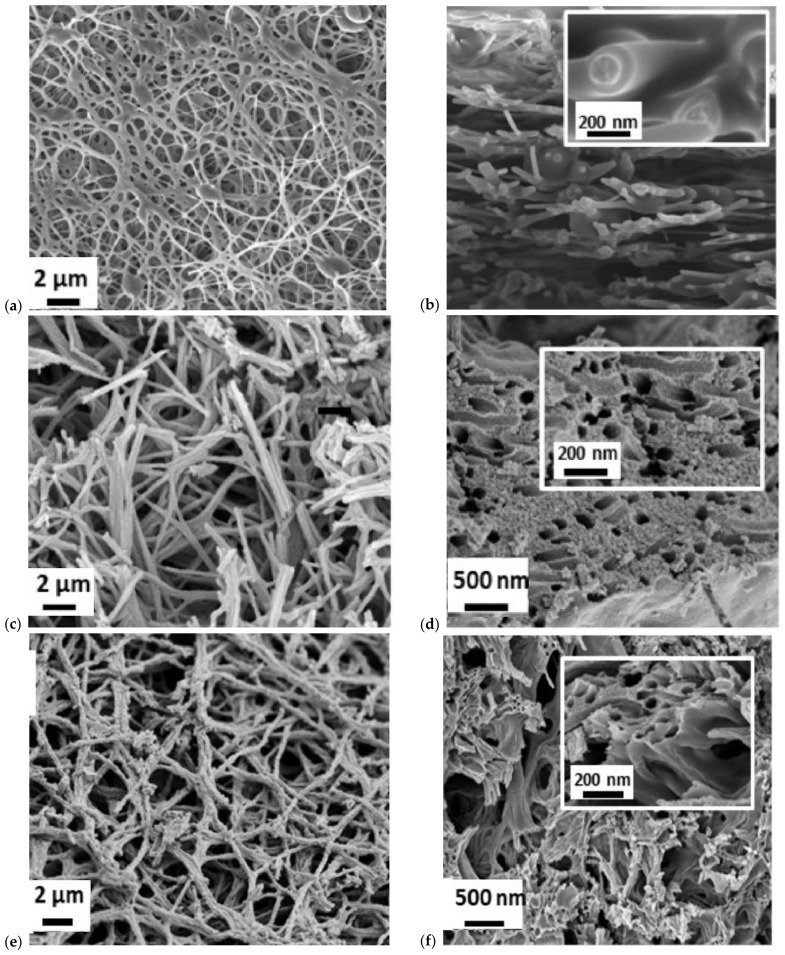
SEM images of THN at various magnifications: (**a**) THN-400 at 5 k; (**b**) THN-400 at 10 k; (**c**) THN-500 at 5 k; (**d**) THN-500 at 10 k; (**e**) THN-600 at 5 k; (**f**) THN-600 at 10 k (reproduction from open access ref. [18]).

**Figure 14:**
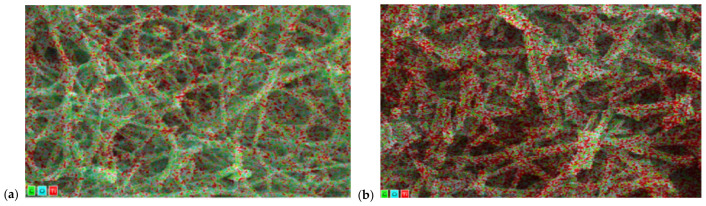
Mapping of elements C (green), O (blue), and Ti (red). (**a**) PAN/TiO_2_ HFs. (**b**) THFs after calcination. Reproduced from open access Ref. [18].

A combined template and electrospinning route was obtained by Jafri et al. [18], the schematic of which is depicted in Figure 12. Using 8 wt.% PAN (average MW ≈ 150,000) dissolved in dimethylformamide (DMF) as a dope solution, they prepared nanofibers through electrospinning conducted at a flow rate of 1 mL/h, 15 cm distance from the needle tip to the collector, and 12 kV voltage. The as-spun PAN nanofibers formed a template which, after drying, was dip-coated in a TiO_2_ sol–gel consisting of 10 mL TTIP, 10 mL acetic acid, 100 mL DW, 1 mL nitric acid, and HNO_3_, with a 5 mm/s immersion/withdrawal speed. The PAN/TiO_2_ fibers, after drying at 90 °C for 2 h, were calcined in air at 400, 500, and 600 °C for 4 h, with the samples being denoted as THN-400, THN-500, and THN-600, accordingly. Their morphology is shown in the field emission SEM images of Figure 13, together with their observed pore size diameter distribution. The calcination temperature was found to significantly influence the morphology, as it not only determines the fibrous morphology (decreasing diameters as the calcination temperature increased), but also the formation of a hollow structure, which was most apparent in THN-600 but not observed in THN-400. The element mapping of PAN/TiO_2_ nanofibers through energy-dispersive X-ray (EDX) showed the Ti, O, and C weight percentages being 35%, 55.1%, and 9.9% and changing to 52.1%, 44.9%, and 2.9% after the calcination (Figure 14). The decrease in the carbon percentage indicates organic decomposition during the calcination process, thus resulting in the formation of the nanofibers’ hollow structure.

The electrospun TiO_2_ hollow nanofibers (THNs) formed a self-standing film after Buchner filtering, which was tested in a batch reactor for the photodegradation of BPA (10 mg/L) under a UV lamp (3.0 mW/cm^2^, λ = 312 nm, 30 W) positioned 15 cm away. HFs calcinated at 600 °C successfully degraded 71.5% of BPA, with 0.75 g/L being the best performing dosage.

## 4. Conclusions

A concise overview of the fabrication techniques used for photocatalytic HFs and photocatalytic HFMs took place. HFMs are mostly prepared through the spinning/phase inversion route, as the gradual substitution of the polymer’s good solvent by the polymer nonsolvent (usually water) gifts an HF with the significant pore structure that enables it to act as a separative membrane when operated under flow conditions. HFMs may bear the photocatalyst either embedded in their matrix (mixed matrix) HFs, or coated on top afterwards and being more accessible to the light. In both cases, it is the nature of the application that turns a porous HF into an HFM, not a special structural characteristic. On the other hand, HFs, acting solely as photocatalyst immobilizers, do not need a porous network for filtration reasons, and can be, therefore, synthesized also by the template or electrospinning method. Compared to flat sheets, their configuration brings advantages like the enhanced active area, which provides abundant photocatalytic sites for interface reactions, and the hollow inside space, which is beneficial for light harvesting and multiple reflecting/scattering effects. In any case, these multistructures produce conducive heterojunctions, accelerating the quick and direct movement of the built-in electric field towards the demanded separation of photoexcited charges.

In terms of efficiency, a direct comparison of the fabricated photocatalytic HFs/HFMs is not achievable, as a uniform protocol or standard way of evaluating the efficiency of a catalyst does not exist. Even in cases of simple liquid phase batch experiments of heterogeneous catalysis, every group uses a different real or model pollutant, different catalyst quantity, and different illumination system (UV/visible light, watt of the lamps, emission λ_max_ and distance from the catalyst), with a different irradiation density reaching the surface of the catalyst. This makes every case study a unique one, almost incomparable with others. This problem expands in the case of HFMs, which perform under flow conditions. There are articles using HFMs for simultaneous pollutant filtration and degradation, which require specially designed photocatalytic reactors. These reactors are usually custom-made to serve the needs of each specific application, something that, practically, turns every single experiment into a unique and specialized one, making comparison attempts almost impossible. There are also articles which apply HFMs solely for filtering separation and irradiate them statically (batch conditions) for photocatalytic antifouling/self-cleaning purposes, targeting the reinstatement of the initial membrane’s water permeability value.

Consequently, a comparison of the general characteristics (advantages and disadvantages) of the fabrication techniques is attempted in Table 5.

To underline the encountered literature gaps within the field, one could observe that more than half of the articles do not mention any photocatalyst leaching or long-term membrane durability issues. Others, although they mention long-term stability of their HFs/HFMs, do not present any corresponding experimental data. Information about the number of successive operational cycles or degradation of mechanical properties vs. irradiation time is of great importance because exposure to radiation is expected not only to have a catalytic influence on the pollutant molecules but also on the polymeric body of the fiber. This aspect was covered only by Rakkapao et al. [30], who reported the photodegradative vanishing of the α-crystalline PVDF phase in alignment with the reduction in mechanical properties due to UV exposure. Total-ceramic fibers, which have lost their polymeric content after a calcination/sintering process, are more pronounced to prove photocatalysis unaffected. Table 6 gathers all the information provided in the presented articles, regarding HFs/HFMs stability/reusability.

Apart from the long-term mechanical strength of the HFs/HFMs, another factor indicative of the scalability and industrial applicability of a reactor system is its energy efficiency. Data of this kind, although valuable, are generally rare in the literature and, in this case, totally absent. Adding energy efficiency data in future articles on the field is highly recommended. Future studies could also emphasize exposing samples to solar light testing systems, reducing, therefore, the expense for artificial irradiation.

## Figures and Tables

**Table 1:** Comparative presentation the articles describing the mixed matrix single-layer and dual-layer spinning/phase inversion/sintering fabrication of HFs/HFMs.

Polymer/ Photocatalyst/Method (SL*/DL*)	BET (m^2^/g)	Pore Size (nm)	Light Source	Application (Batch/Flow)/Contaminant/ Parameters	Efficiency	Ref.
Alginate/SL TiO_2_/wet/wet + sc-CO_2_ drying	▪ Nonporous: 33 ▪ porous sc-CO_2_: 94	▪ Nonporous: 12.4 ▪ porous sc-CO_2_: 9.4	4 UVA lamps 9 W, 315–380 nm, λmax 365 nm, 2.1 mW/cm^2^	Batch/MO 20 mM, HFs 10 cm, 31 cm^2^, TiO_2_ 3.6 g/L	▪ Nonporous: 90% in 325 min ▪ Porous sc-CO_2_: 90% in 220 min	[12]
Alginate/SL CuO-TiO_2_/wet/wet	49.2	35.1	4 UVA, 15 W, 350–390 nm, 0.5 mW/cm^2^, 5 cm distance	Batch/MO 6.3, 10, 12, 15, 18 ppm (O_2_), 2.5 g/L HFs	▪ 6.3 ppm, 67.8%, 2 h ▪ 18 ppm 60.5%, 3 h	[13]
Alginate/SL/ Cu/CuO-TiO_2_/wet/wet	61.9	10–70	4 UVA, 15 W, 350–390 nm, 0.5 mW/cm^2^, 5 cm distance	Batch/MO 6.3, 10, 12, 15, 18, 24 ppm (O_2_), 75 mg HFs	Most concentrations: 65% degradation in 180 min	[14]
Alginate/SL TiO_2_ + C_60_ TiO_2_ + CNTs TiO_2_ + GO/wet/wet	4% GO-TiO_2_: 110		near-UV/Vis 350 < λ, 50 mW/cm^2^, VIS: λ < 430 nm, 6 mW/cm^2^	Batch/MO: 7.5 mL, 10 ppm, 0.5 g/L, DP: 7.5 mL, 100 ppm, 1 g/L	4%GO-TiO_2_ UV: ▪ MO: k = 7.7 × 10^−3^ min^−1^ ▪ DP: k = 3.4 × 10^−3^ min^−1^	[27]
Alginate/PVP/CNTs/TiO_2_/needle			Xe 350 W	200 ppm NO, 20 mg catalyst	44.3% NO reduction	[46]
PEI/SL TiO_2_/sintering at 700 °C and 900 °C	700 °C: 15.3, 900 °C 5.0	700 °C: 7.5, 900 °C: 5.6		Batch/AO7, 25 mL, 20 ppm, 50 mg HFs, area: 1.5 × 10^−4^ m^2^	▪ 66.7% at 700, 12.2 L/m^2^h ▪ 90.2% under UV	[35]
PEI/SL TiO_2_/sintering at 600, 700, 900 °C	600 °C: 63.3, 700 °C: 15.3	600 °C: 2.82, 700 °C: 28.9	UVA lamps 8 W, 330–370 nm, 0.17 mW/cm^2^	Flow/antifouling/self-cleaning, 50 mg HFs, area: 1.5 × 10^−4^ m^2^	Permeability 7.9 L/m^2^h under UV	[36]
PMIA/SL/ TiO_2_/dry/wet		132–256 (from SEM)	UV light, 10 cm distance	Flow/125 mL, 10 mg/L MB, 20 cm HFMs	132.6 L/m^2^h at 4.76 wt% TiO_2_	[33]
PES/SL TiO_2_/dry/wet			UVA 18 W, 365 nm, 0.6 mW/cm^2^, 12 cm	Batch/10 mol/L MB, 3 HFs, 20 cm, area 0.0036 m^2^	Qualitative MB decolorization in 5 h	[7]
PES/SL/Nb_2_O_5_ GO-Nb_2_O_5_/wet/wet/1200 °C			UVc, 150 W, 100 mW/cm^2^, 10 cm	Batch/MB 10 mg/L, 6 cm HFs, pH 11, 57.4 Nb_2_O_5_ mg/L	In 210 min HFs: ▪ Nb_2_O_5_: 66.4%, ▪ GO-Nb_2_O_5_ 100%	[5]
PVDF/SL TiO_2_/wet/wet	10.11		UVc, 150 W, 100 mW/cm^2^, 10 cm distance	Batch/MB 400 mL, 10 ppm, pH 11, HFs 6 cm, TiO_2_ 57.5 mg/L	MB 100% degradation in 210 min	[6]
PVDF/SL/NPs-TiO_2_/dry/wet air gap 24 cm		0.135 μm	2 UVA, 18 W, 365 nm, at 6 cm, 2.7 mW/cm^2^	Flow/MB 250, 10 μm/L, 3 HFs, 20 cm, area 0.0036 m^2^	Rejection 97%	[8]
PVDF/TiO_2_/DL/dry/wet, 10 cm air gap		164–264	UVA, 8 W, 365 nm, 0.33 mW/cm^2^	Batch/NP 100 ppm, 4 h	NP decomposition within 150 min (K_app_ = 0.0173 min^−1^)	[26,53,54]
PVDF/ TiO_2_/DL dry/wet 10 cm air gap		164–264	UVA 8 W, 365 nm, 0.33 mW/cm^2^	Flow, NP antifouling	0.2 TiO_2_/PVDF: NP flux 25 L/m^2^h	[37,54]
PVDF/ TiO_2_/DL dry/wet 10 cm air gap		164–264	UV, 40 W, 254 nm, 50 mW/cm^2^	Batch/90 DLHFs, 70 cm, TiO_2_ 57 mg/L, 200–400 μg/L	Metoprololand trimethoprim k > 0.08 min^−1^ > than P25	[29,53,54]
PVDF TiO_2_-WO_3_@GO/DL			VIS (LED 100 W)	Flow/antifouling/20 HFMs, 10 cm, 5 cycles	Initial/final permeability: 99.5/94 L/m^2^h	[40]
PVDF Cu_2_O/DL		101–129	VIS (LED 100 W)	Flow (water), batch: BPA 10 mg/L	BPA 75% in 360 min	[20]
PVDF CuO/VS_4_/DL		20–28.4	VIS (LED 100 W)	Flow/BPA, 500 mL, 1 mg/L, 10 HFMs, 20 cm, 3.52 × 10^–3^ m^2^	BPA degradation + rejection: 3.75%, 4 h	[21]
PVDF/MoO_3_/ZnO/GO/DL			VIS (LED 100 W)	Batch, 10 L, EDCs 1 μg/Lmg/L	53–100% in 240 min	[25]
PVDF/ 3Al_2_O_3_*2SiO_2_/TiO_2_ DL			UV 6 W	4 g catalyst, batch: COD and color/flow: POME rejection,	45% COD, 52.4% color, POME rejection 54.5%	[31]
PVDF/TiO_2_/PVP/SL		0.5 μm	60 W UV	Flow, 10 HFMs, 20 cm, 0.3–20 mg/L	60% removal carbaryl pesticide	[30]

SL* = single layer, DL* = dual layer.

**Table 2:** Comparative presentation of the articles describing external coating of photocatalyst HFs/HFMs.

Substrate	Photocatalyst/ Deposition	Light Source	Application (Batch/Flow)/Parameters	Efficiency	Ref.
PSf HFs (spinning)	Ag@TiO_2_/ dip coating	100 W Xe lamp (visible light).	Batch/BPA 10 mg/L, 100 mL, HFs 8 cm length	88% of degradation in 270 min	[22,23]
PES/Al_2_O_3_ HFMs (dry/wet spinning), sintering 1400 °C	CuO/CeO_2_/dip coating	UV	Flow/antifouling	Flux from 19 to 30.5 L/m^2^hbar	[38]
PVDF HFMs/CNTs	WO_3_/g-C_3_N_4_/CNTs	Xe 350 W	Flow/MB	89.47% after 4 h.	[34]
PSf HFs (spinning)	N-TiO_2_/ dip coating	visible light	humic acid (model foulant), 20 mg/L, pH = 7	100% initial pure water permeate	[41]
PES/Al_2_O_3_ (spinning/sintering 1300 °C 3 h	N-TiO_2_/dip coating	LED (white, blue, UV)	Flow reactor, (30, 32, 36, 42 HFMs), 15 min	100% gas NH_3_ degradation	[44]
PES commercial/HFMs	0.1% *w*/*v* TiO_2_ spray, filtration coating	Simulated solar light, 1500 W.	Flow/MB and CHD, 4 HFMs 10 cm, area 7.22 cm^2^, 40–60 kPa	MB 30%, CHD 40%	[9]
PES/Al_2_O_3_ HFMs (spinning)/sintering 1400 °C	Electrospinning externally PAN + 0.8 wt.% of g-C_3_N_4_	UV irradiation (30 W lamp, peak at 312	Flow/antifouling/self-cleaning NF-g-C_3_N_4_/Al_2_O_3_:	before:816 L/m^2^h, after 180 min UV: 577 L/m^2^h	[39]
Al_2_O_3_ PES/500 °C	S-TNTs (100 mL, 0.2 g/L)/vacuum filtration	Xenon, 15 cm, 220 mW/cm^2^	Batch MO solution (10 mg/L, 50 mL, pH = 7), Flow oil/OPW	99.89% MO, flux recovery: 85.8% oil, 57.6% OPW	[43]

**Table 3:** Comparative presentation of the articles describing the electrospinning-produced photocatalytic HFs/HFMs.

Polymer/Photocatalyst (Precursor)/Calcination	BET (m^2^/gr)	Fiber Diameter/Wall Thickness	Light Source	Contaminant/Operating Parameters	Efficiency	Ref.
PVP/TiO_2_ (TBOT), 500 °C (1 °C/min, 3 h, air)	27.2	215 nm/100 nm	300 W Xe λ > 320 nm	RhB 10 mg/L	RhB 99.5% (1 h), 499.1 μmol H_2_/gh	[16]
PVP (PS)/TDIP/500 °C		425.5–353.5 nm/-	8 UV, 352 nm, 10 W	MB	k = 0.0772 min^−1^	[11]
PVP/TiBuT/500 °C	51.28	1.45–3.95 μm/-	2 UV_A_ 6 W, 10 W/m^2^, 365 nm	3 L/min, 1 ppm NO, RH 50%	66.2%.	[47]
In situ growth of MOFs UiO-66 on Fe-TiO_2_ HF	135.77	511.3 nm/-	UV/Vis: 405/550 nm, 1.10/2.59 mW/cm^2^	Acetaldehyde	30–40% RH: UV 69.8%, Vis 37.9%, UV/Vis 73.3%	[45]
Pluronic/TiO_2_ (TiBut)/400 °C (4 h, air, 0.5 °C/min)	208	0.1–4 μm/60–500 nm	UV, 125 W, λ_max_ = 365 nm	MB (100 mL, 2 mg/L)	100% decolonization of MB, 40 min	[1]
PMMA/ZnO-SnO_2_-Zn_2_SnO_4_ (800 °C), Zn_2_SnO_4_ (1000 °C)	21.7/37.67	diameter 700 nm −2 μm	UV, 8 W, λ_max_ = 365 nm	BPA 10^−5^ M, 100 mg HFs	64% Phenol, 49% BPA	[10]

**Table 4:** Comparative parameters for the template-method-derived photocatalytic HFs.

Template/Photocatalyst (Precursor)/Sintering Temperature	Surface Area (m^2^/g)	HF Diameter	Light Source	Contaminant/Operating Parameters	Efficiency	Ref.
Cotton/TiO_2_ (TBT)/450–750 °C	6.5–11.5		800 W Xenon (70.8 × 10^3^ lux)	MB 50 mL, 10 mg/L, 50 mg HFs	MB 96% in 2 h	[2]
Kapok/Fe-TiO_2_ (TiBut)/450 °C		20–30 μm.	white LED irradiation, 20 W	MB 50 mL, 5 ppm	1% Fe-HF: MB 67% in 6 h	[3]
Ca-alginate fibers/In_2_O_3_/ZnIn_2_S_4_	49.3–55.7		Xe 300 W, 173 mW/cm^2^)	TEOA, Pt cocatalyst	2.18 mmol/g/h H_2_ evolution	[48]
Kapok/Au-TiO_2_ (TBT)/450 °C	Au/THF: 62.1		Vis–LED lamp (50 W, Sylvania)	MB 10 ppm 10 cm away, 1 g/L	88% MB degradation, 8 h	[4]
Kapok/TiO_2_ (TiBut)/450–750 °C	450 °C: 42.4, 750 °C: 14.7		UV lamp, 6 W, λ 365 nm	GMX 100 mL,10 ppm	450 °C THFs k = 1.39 × 10^−3^ min^−1^	[28]

**Table 5:** Comparative presentation of the advantages and disadvantages of the fabrication categories described.

Fabrication Technique	Advantages	Disadvantages
Mixed matrix Spinning/Phase Inversion	Polymer/additive options Fabrication of both HFs and HFMs Adjustable dimensions (OD/ID) Adjustable (considerable) length High porosity/pore size control High production rate Dual layer possibility No leaching of the photocatalyst	Requires solvent Expensive equipment required
External Photocatalyst coating	Many substrate options Fabrication of both HFs and HFMs High production rate	leaching of the photocatalyst
Electrospinning	Easiness of size/structure control Simple/cheap equipment Polymer/additive options Adjustable (considerable) length	Low production rate No porosity/only for HFs Only ultra-fine fabric
Template	Cheap natural templates High production rate	No porosity/only for HFs High-energy-demanding sintering for template removal Short length

**Table 6:** Cumulative stability/reusability information provided by the articles.

Stability/Reusability Information Provided	Ref.
After 4 cycles, 9% decrease in performance, catalyst leaching, decrease in Au content in EDS.	[4]
After 4 operational cycles (240 min), the fibers were stable.	[5]
2 h UV (0.6 mV/cm^2^) damaged the selective layer.	[7]
Long-term stability under UV by adding the hydrophilic pore-former PEG.	[8]
After 6 operational cycles, SEM shows unaffected microstructure.	[10]
HFs damaged after 5 cycles (21 h) UV.	[12]
After 4 consecutive cycles, resistance to attrition + retain high activity.	[13]
After 5 operational cycles (180 min), the system kept of 92% the initial performance.	[14]
3 cycles.	[16]
After 5 cycles, 80% of the initial weight remained and 54.5% of the initial performance.	[18]
No leaching.	[20]
After 3 cycles, 74.05% regeneration efficiency.	[21]
After 3 cycles, 71.33% regeneration efficiency.	[24]
After 3 cycles, 74.02% regeneration efficiency.	[25]
70% of performance kept for several cycles.	[27]
5 cycles.	[29]
Photodegradation under UV.	[30]
Stability test performed; no results presented.	[31]
The sample with 4.76% TiO_2_ had the best long-term stability + antifouling resistance.	[33]
After 5 cycles of 7 h UV irradiation each, SEM confirmed no leaching.	[40]
After 6 days UV: • PSf HFM 171% of the initial flux, • photocatalytic HFM was stable.	[41]
UV damages the membrane and increases the flux.	[42]
Stable after 12 cycles of 1 h photocatalytic operation +1 h static photocatalytic self-cleaning.	[43]
5 cycles of performance: 1st: 74.4%, 2nd: 72.1%, 3rd: 69.4%, 4th: 73.2%, 5th: 72.6%.	[45]
After 4 cycles, 4% decrease in activity + no leaching confirmed by SEM and XRD.	[46]
SEM before and after irradiation, shows good stability.	[48]

## Data Availability

No new data were created or analyzed in this study. Data sharing is not applicable to this article.

## Abbreviations

The following abbreviations are used in this manuscript:

HF	Hollow fiber
HFM	Hollow fiber membrane
THF	TiO_2_ hollow fibers
MB	Methylene blue
MO	Methyl orange
AO7	Acid orange 7
DLHF	Dual layer hollow fibers
PEI	Polyethyleneimine
PMIA	Poly(m-phenylene isophthalamide
PES	Polyethersulfone
PSf	Polysulfone
PS	Polystyrene
PAN	Polyacrylonitrile
PVDF	Polyvinylidene fluoride
PVP	Polyvinylpyrrolidone
HPLC	High-performance liquid chromatography
RhB	Rhodamine B
BPA	Bisphenol A
DP	Diphenylamine
OPW	Oilfield-produced water
LDOPA	3-(3,4-dihydroxyphenyl) lalanine
TOC	Total organic carbon
GO	Graphene oxide
CNT	Carbon nanotube
GMX	Gramoxone
NP	Nonyphenol
TBT	Tetrabutyl titanate
POME	Palm Oil Mill Effluent

## References

[1] Zhan S., Chen D., Jiao X., Tao C. (2006). Long TiO_2_ Hollow Fibers with Mesoporous Walls: Sol-Gel Combined Electrospun Fabrication and Photo-catalytic Properties. J. Phys. Chem. B.

[2] Zheng T., Tian Z., Su B., Lei Z. (2012). Facile Method To Prepare TiO_2_ Hollow Fiber Materials via Replication of Cotton Fiber. Ind. Eng. Chem. Res..

[3] Wongcharoen S., Chokradjaroen C., Panomsuwan G. (2020). Effect of Fe doping on the photocatalytic activity of TiO_2_ hollow fibers under LED light irradiation. Mater. Sci. Eng..

[4] Panomsuwan G., Wongcharoen S., Chokradcharoen C., Tipplook M., Jongprateep O., Saito N. (2022). Au nanoparticle-decorated TiO_2_ hollow fibers with enhanced visible-light photocatalytic activity. RSC Adv..

[5] de Paulo Ferreira E., Ribeiro S.R.F.L., Sousa T.S.E., Cardoso V.L., Reis M.H.M. (2025). Surface decoration of Nb_2_O_5_ hollow fibers by graphene oxide for enhanced photocatalytic degradation of methylene blue. J. Am. Ceram. Soc..

[6] Abdullah N., Ayodele B.V., Nurdiyan W., Mansor W., Abdullah S. (2018). Effect of Incorporating TiO_2_ Photocatalyst in PVDF Hollow Fibre Mem-brane for Photo-Assisted Degradation of Methylene Blue. Bull. Chem. React. Eng. Catal..

[7] Simone S., Galiano F., Faccini M., Boerrigter M.E., Chaumette C., Figoli E.D.A. (2017). Preparation and Characterization of Polymeric-Hybrid PES/TiO_2_ Hollow Fiber Membranes for Potential Applications in Water Treatment. Fibers.

[8] Galiano F., Song X., Marino T., Boerrigter M., Saoncella O., Simone S., Faccini M., Chaumette C., Drioli E., Figoli A. (2018). Novel Photocatalytic PVDF/Nano-TiO_2_ Hollow Fibers for Environmental Remediation. Polymers.

[9] Chakraborty S., Loutatidoua S., Palmisano G., Kujawa J., Mavukkandy M.O., Al-Gharabli S., Curcio E., Arafat H.A. (2017). Photocatalytic hollow fiber membranes for the degradation of pharmaceutical compounds in wastewater. J. Environ. Chem. Eng..

[10] Dasa P.P., Roya A., Tathavadekar M., Devia P.S. (2017). Photovoltaic and photocatalytic performance of electrospun Zn_2_SnO_4_ hollow fibers. Appl. Catal. B Environ..

[11] Nguyen T.T.H., Nguyen H.H., Cho Y.S. (2023). Fabrication of hollow titania fibers by electro-spinning for photocatalytic degradation of organic dyes. J. Korean Ceram. Soc..

[12] Papageorgiou S.K., Katsaros F.K., Favvas E.P., Romanos G.E., Athanasekou C.P., Beltsios K.G., Falaras O.I.T.P. (2012). Alginate fibers as photo-catalyst immobilizing agents applied in hybrid photocatalytic/ultrafiltration water treatment processes. Water Res..

[13] Theodorakopoulos G.V., Romanos G.E., Katsaros F.K., Papageorgiou S.K., Kontos A.G., Spyrou K., Beazi-Katsioti M., Falaras P. (2021). Struc-turing efficient photocatalysts into bespoke fiber shaped systems for applied water treatment. Chemosphere.

[14] Theodorakopoulos G.V., Papageorgiou S.K., Katsaros F.K., Romanos G.E., Beazi-Katsioti M. (2024). Investigation of MO Adsorption Kinetics and Photocatalytic Degradation Utilizing Hollow Fibers of Cu-CuO/TiO_2_ Nanocomposite. Materials.

[15] Theodorakopoulos G., Athanasekou C., Romanos G.E., Papageorgiou S.K. (2020). Current photocatalytic systems for intensified water purifica-tion applications. Handbook of Smart Photocatalytic Materials, Fundamentals, Fabrications, and Water Resources Applications.

[16] Hou H., Shang M., Wang L., Li W., Tang B. (2015). Efficient Photocatalytic Activities of TiO_2_ Hollow Fibers with Mixed Phases and Mesoporous Walls. Sci. Rep..

[17] Thiruvenkatachari R., Kwon T.O., Moon S. (2005). Application of Slurry Type Photocatalytic Oxidation-Submerged Hollow Fiber Microfiltration Hybrid System for the Degradation of Bisphenol A (BPA). Sep. Sci. Technol..

[18] Jafri N.N.M., Jaafar J., Aziz F., Salleh W.N.W., Yusof N., Othman M.H.D., Rahman M.A., Ismail A.F., Rahman R.A., Khongnakorn W. (2022). Development of Free-Standing Titanium Dioxide Hollow Nanofibers Photocatalyst with Enhanced Recyclability. Membranes.

[19] Kamaludin R., Rasdi Z., Othman M.H.D., Kadi S.H.S.A., Nor N.S.M., Khan J., Zain W.N.I.M., Ismail A.F., Rahman M.A., Jaafar J. (2020). Visi-ble-Light Active Photocatalytic Dual Layer Hollow Fiber (DLHF)Membrane and Its Potential in Mitigating the Detrimental Effects of Bisphenol A in Water. Membranes.

[20] Noor S.H.M., Othman M.H.D., Khongnakorn W., Sinsamphanh O., Abdullah H., Puteh M.H., Kurniawan T.A., Zakria H.S., El-badawy T., Ismail A.F. (2022). Bisphenol A Removal Using Visible Light Driven Cu_2_O/PVDF Photocatalytic Dual Layer Hollow Fiber Membrane. Membranes.

[21] Zakria H.S., Othman M.H.D., Borhamdin S., Ismail N.J., Rahman M.A., Jaafar J., Puteh M.H., Yahaya N.K.E.M., Idris A., Kurniawan T.A. (2025). PVDF/CuO–VS4 Dual-Layer Hollow Fiber Photocatalytic Membrane for Bisphenol A Removal with Energy Storage Capability. Korean J. Chem. Eng..

[22] Shareef U., Othman M.H.D., Ismail A.F., Jilani A. (2019). Facile removal of bisphenol A from water through novel Ag-doped TiO_2_ photocatalytic hollow fiber ceramic membrane. J. Aust. Ceram. Soc..

[23] Shareef U., Waqas M. (2020). Bisphenol A Removal through Low-Cost Kaolin-Based Ag@TiO_2_ Photocatalytic Hollow Fiber Membrane from the Liquid Media under Visible Light Irradiation. J. Nanomater..

[24] Zakria H.S., Othman M.H.D., Kamaludin R., Jilani A., Omar M.F., Ayub M., Saidin M.A.R., Kurniawan T.A., Hashim N., Yahaya N.K.E.M. (2023). Removal of bisphenol A from synthetic and treated sewage wastewater using magnetron sputtered CuxO/PVDF thin film photocatalytic hollow fiber membrane. J. Water Process Eng..

[25] Zakria H.S., Borhamdin S., Ismail N.J., Peechmani P., Moslan M.S., Othman M.H.D., Rahman M.A., Jaafar J., Puteh M.H., Rajamohan N. (2024). Novel self-cleaning PVDF/MoO_3_/ZnO/GO dual layer hollow fiber photocatalytic membrane with excellent photocatalytic performance of EDCs removal and energy storage capability. J. Mem. Sci..

[26] Dzinun H., Othman M.H.D., Ismail A.F., Puteh M.H., Rahman M.A., Jaafar J. (2016). Photocatalytic degradation of nonylphenol using co-extruded dual-layer hollow fibre membranes incorporated with a different ratio of TiO_2_/PVDF. React. Funct. Polym..

[27] Pastrana-Martínez L.M., Morales-Torres S., Papageorgiou S.K., Katsaros F.K., Romanos G.E., Figueiredo J.L., Faria J.L., Silva A.M.T. (2013). Photocatalytic behaviour of nanocarbon–TiO_2_ composites and immobilization into hollow fibres. Appl. Catal. B Environ..

[28] Wongcharoen S., Panomsuwan G. (2018). Easy synthesis of TiO_2_ hollow fibers using kapok as a biotemplate for photocatalytic degradation of the herbicide paraquat. Mater. Lett..

[29] Paredes L., Murgolo S., Dzinun H., Othman M.H.D., Ismail A.F., Carballa M., Mascolo G. (2019). Application of immobilized TiO_2_ on PVDF dual layer hollow fibre membrane to improve the photocatalytic removal of pharmaceuticals in different water matrices. Appl. Catal. B Environ..

[30] Rakkapao N., Khongnakorn W., Jeenderm S., Tepkeaw N., Khongcharoenthin S., Thammakhet-Buranachai C., Jaafar J. (2025). Titanium dioxide incorporated in polyvinylidene fluoride hollow fiber membrane for carbaryl removal and degradation. Int. J. Environ. Sci. Technol..

[31] Raji Y.O., Othman M.H.D., Puteh M.H., Jasman S.M., Jaafar J., Rahman M.A., Ismail A.F., Salisu M., Heng J., Gunawan T. (2026). Photocatalytic treatment of final discharge palm oil mill effluent (POME) using dual-layer hollow fiber ceramic membranes with TiO_2_-embedded mullite: Performance evaluation and mechanistic insights. Mater. Sci. Eng. B..

[32] Athanasekou C. (2026). Photocatalytic Applications of Hollow Fibers and Hollow Fiber Membranes. Photochem.

[33] Hwang G.-I., Lee S., Lee J.-H., Moon J., Shin Y.-R., Baik D.H., Jee M.H., Jeong Y.G. (2025). TiO_2_-incorporated poly(m-phenylene isophthalamide) hollow fiber membranes for superior ultrafiltration, mechanical strength, photocatalytic activity in water treatment. Chem. Eng. J..

[34] Geng S., Zhao X. (2026). The curtain hollow fiber photocatalytic membrane module fabricated from WO_3_/g-C_3_N_4_/CNTs composite material exhib-its the filtration-adsorption-photocatalysis synergistic effect. J. Photochem. Photobiol. A Chem..

[35] Zhang X., Wang D.K., Lopez D.R.S., da Costa J.C.D. (2014). Fabrication of nanostructured TiO_2_ hollow fiber photocatalytic membrane and applica-tion for wastewater treatment. Chem. Eng. J..

[36] Wang D.K., Elma M., Motuzas J., Hou W.-C., Xie F., Zhange X. (2017). Rational design and synthesis of molecular-sieving, photocatalytic, hollow fiber membranes for advanced water treatment applications. J. Membr. Sci..

[37] Dzinun H., Othman M.H.D., Ismail A.F., Puteh M.H., Rahman M.A., Jaafar J., Adrus N., Hashim N.A. (2018). Antifouling Behavior and Separation Performance of Immobilized TiO_2_ in Dual Layer Hollow Fiber Membranes. Polym. Eng. Sci..

[38] Abdullah N., Rahman M.A., Othman M.H.D., Ismail A.F., Jaafar J., Aziz A.A. (2016). Preparation and characterization of self-cleaning alumina hollow fiber membrane using the phase inversion and sintering technique. Ceram. Int..

[39] Alias N.H., Jaafar J., Samitsu S., Matsuurae T., Ismail A.F., Othman M.H.D., Rahman M.A., Othman N.H., Abdullah N., Paiman S.H. (2019). Photocatalytic nanofiber coated alumina hollow fiber membranes for highly efficient oilfield produced water treatment. Chem. Eng. J..

[40] Samuel O., Khan A.U., Kamaludin R., Othman M.H.D., Kurniawan T.A., Imtiaz A., Al-Ogaili M.F., Usman J., Muhammad M.S., Abdul-kareem B. (2024). Dual layer hollow fiber photocatalytic membrane based on TiO_2_-WO_3_@GO composite with catalytic memory and enhanced anti-fouling and self-cleaning properties for oilfield-produced water treatment. Chem. Eng. J..

[41] Wan P., Zhang Z., Deng B. (2019). Photocatalytic Polysulfone Hollow Fiber Membrane with Self-Cleaning and Antifouling Property for Water Treatment. Ind. Eng. Chem. Res..

[42] Wan P., Wu T., Shi S., Zhao Q., Deng B. (2020). Self-cleaning and antifouling polyvinylidene difluoride hollow fiber membrane enabled by visible light irradiation for water treatment. Desalin. Water Treat..

[43] Fu W., Zhang L., Wu W., An H., Feng M., Sun M., Zhao Y., Chen L. (2025). S-doped TiO_2_ nanotubes decorated whisker mullite-based hollow fiber membrane with anti-fouling and photocatalytic self-cleaning properties for high-efficient separation of emulsified oily wastewater. Sep. Purif. Technol..

[44] Magnone E., Hwang J.Y., Shin M.C., Zhuang X., Lee J.I., Park J.H. (2022). Al_2_O_3_-Based Hollow Fiber Membranes Functionalized by Nitro-gen-Doped Titanium Dioxide for Photocatalytic Degradation of Ammonia Gas. Membranes.

[45] Byun J., Sheraz M., Lee B.-S., Kim J. (2026). In situ growth of UiO-66 on Fe-TiO_2_ hollow fiber for adsorption and photocatalytic degradation of acet-aldehyde under UV and visible light. Sep. Purif. Technol..

[46] Zhang Z., Guo Y., Long F., Zhang X., Wang J., Ren Y. (2025). Continuous fabrication of hollow photocatalytic CNT/TiO_2_/calcium alginate microfibers via needle-based microfluidic device for environmental remediation in NO removal. J. Environ. Chem. Eng..

[47] Kim J. (2022). Hollow TiO_2_/Poly (Vinyl Pyrrolidone) Fibers Obtained via Coaxial Electrospinning as Easy-to-Handle Photocatalysts for Effective Ni-trogen Oxide Removal. Polymers.

[48] Lu P., Liu K., Liu Y., Ji Z., Wang X., Hui B., Zhu Y., Yang D., Jiang L. (2024). Heterostructure with tightly-bound interface between In_2_O_3_ hollow fiber and ZnIn_2_S_4_ nanosheet toward efficient visible light driven hydrogen evolution. Appl. Catal. B Environ..

[49] Athanasekou C., Moustakas N., Morales-Torres S., Pastrana-Martínez L.M., Figueiredo J.L., Faria J.L., Silva A.M.T., Dona- Rodriguez J.M., Romanos G.E., Falaras P. (2015). Ceramic photocatalytic membranes for water filtration under UV and visible light. Appl. Catal. B Environ..

[50] Athanasekou C., Morales-Torres S., Likodimos V., Romanos G.E., Pastrana-Martinez L.M., Falaras P., Faria J.L., Figueiredo J.L., Silva A.M.T. (2014). Prototype composite membranes of partially reduced graphene oxide/TiO_2_ for photocatalytic ultrafiltration water treatment under visible light. Appl. Catal. B Environ..

[51] Romanos G.E., Athanasekou C., Likodimos V., Aloupogianni S.P., Falaras P. (2013). Hybrid Ultrafiltration/Photocatalytic Membranes for Effi-cient Water Treatment. Ind. Eng. Chem. Res..

[52] Pradhana E.A., Elma M., Othman M.H.D., Huda N., Ul-haq M.D., Rampun E.L.A., Rahma A. (2021). The Functionalization Study of PVDF/TiO_2_ Hollow Fibre Membranes Under Vacuum Calcination Exposur. J. Phys. Conf. Ser..

[53] Kamaludin R., Othman M.H.D., Kadir S.H.S.A., Rahman M.A., Jaafar J. (2017). The morphological properties of photocatalytic TiO_2_/PVDF dual layer hollow fiber membrane for endocrine disrupting compounds degradation. Malays. J. Anal. Sci..

[54] Dzinun H., Othman M.H.D., Ismail A.F., Puteh M.H., Rahman M.A., Jaafar J. (2015). Morphological study of co-extruded dual-layer hollow fiber, Membranes incorporated with different TiO_2_ loadings. J. Membr. Sci..

[55] Dzinun H., Othman M.H.D., Ismail A.F., Puteh M.H., Rahman M.A., Jaafar J. (2017). Performance evaluation of co-extruded microporous dual-layer hollow fiber membranes using a hybrid membrane photoreactor. Desalination.

